# Effect of face shield design on the prevention of sneeze droplet inhalation

**DOI:** 10.1063/5.0044367

**Published:** 2021-03-26

**Authors:** Fujio Akagi, Isao Haraga, Shin-ichi Inage, Kozaburo Akiyoshi

**Affiliations:** 1 Faculty of Engineering, Fukuoka University, 8–19-1 Nanakuma, Jyounan-ku, Fukuoka, Japan; 2 Department of Anesthesiology, Faculty of Medicine, Fukuoka University, 7–45-1 Nanakuma, Jyounan-ku, Fukuoka, Japan

## Abstract

A flow simulation was performed for face shields to investigate whether varying a shield's edge shape could prevent droplets from entering the shield. Face shields with two types of edge shapes were used. The “Type I” shield had small plates mounted on the top and bottom edges of the shield to physically inhibit the sneeze inflow. The “Type II” shield had small brims sticking forward from the shield surface and small plates sticking upward and downward at the top and bottom edges to inhibit the entrainment flow produced by the vortex ring using sneeze flow. We confirmed that the flow characteristics around a face shield can be controlled using the shield's edge shape. In Type I, the entraining flow inside the shield was inhibited by the mounted small plate at the bottom edge, ensuring the inhibiting effect, but not at the top edge. In Type II, the entrained flow inside the shield was inhibited by the mounted brim and small plate at the top edge, ensuring the inhibiting effect, but not at the bottom edge. The effects of the Type II design parameters on the flow characteristics around the face shield were examined. The results indicate that at the top edge, increasing the length of the brim and not mounting the small plate at an incline from the shield surface improves the inhibition effect. At the bottom edge, shortening the length of the brim and mounting the small plate at an incline from the shield surface improves the inhibition effect.

## I. INTRODUCTION

The SARS-CoV-2, which was first confirmed in December 2019, in China has caused a global pandemic, with the number of infected people increasing explosively, and the threat remains. Moreover, in this pandemic, the number of infected people exceeded the number of medical staff and medical equipment in several countries; therefore, many infected people and patients with medical cases unrelated to the coronavirus disease could not receive adequate medical treatment.^1,2^ To maintain the quality of medical treatment in such situations, it is extremely important to ensure that infection prevention measures are followed not only by medical staff but also by all persons.^3,4^ Face shields, which are used to cover the face using a clear plastic shield, and medical surgical masks are being actively used by medical staff to protect the eyes, nose, and mouth—the entry points for viruses—from virus-laden droplets that spread via an infected person breathing, coughing, or sneezing.^5–8^ Recently, the number of people who are using face shields as a substitute for face masks has been increasing in schools, universities, restaurants, and service businesses. This increased adoption is due to benefits such as preventing droplets from attaching directly to the face, being able to see facile expressions, ease of hearing, reusability when washed and disinfected properly, and increased comfort compared to regular masks. However, there is one concern regarding this. As face shields and masks were originally developed to prevent the spread of the wearer's own droplets, they may not be effective in preventing infection when used for any other purpose. Furthermore, there is no guarantee that a face shield will have the same preventive effect as a mask.

In the early stages of the outbreak, little was known about the transmission of the SARS-CoV-2 and the methods for preventing infection, but these are now being clarified because of the work conducted by researchers worldwide.^7–21,25^ Their results have demonstrated that some of the prevention measures that were previously considered to be effective have not been completely effective in preventing the spread of the SARS-CoV-2. For instance, it has been suggested that face shields and masks equipped with exhalation valves cannot provide sufficient protection from infection.^13–19^ Verma *et al.* conducted a flow visualization around a face shield and a mask to evaluate their performance in preventing the spread of aerosol-sized droplets when an infected person uses them for the purpose of protecting others.^13^ They observed that aerosols leaked into the air through gaps in face shields and masks and concluded that it may be desirable to use a high-quality cloth or surgical mask with a plain design. This is also applicable when a face shield or mask is worn to prevent infection from infected persons. For example, if a medical professional wearing a face shield is exposed to droplets when an infected person breathes, coughs, or sneezes in front of the wearer's face, the large droplets may attach to the face shield's surface, thereby providing protection; however, small droplets may move with the airflow and be drawn in through the space between the wearer's face and the face shield.^15,22–28^ Akagi *et al.* have conducted numerical simulations to clarify the details of the airflow around a face shield and its effectiveness in preventing infection when a shield wearer is exposed to a sneeze from a nearby patient.^29^ Such conditions are frequently encountered by medical staff while treating or examining patients. Their results indicate the following.

(1) The high-velocity airflow created by sneezing or coughing generates vortex ring structures, which reach the top and bottom edges of the shield and form a high-velocity entrainment flow.
(2) Vortex rings capture small particles, i.e., sneeze droplets and aerosols, and transport them to the top and bottom edges of the face shield because vortex rings have the ability to transport microparticles.
(3) Some particles (in their simulation, 4.4% of the released droplets) entered inside the face shield and reached the vicinity of the nose.

These results demonstrate that a medical worker wearing a face shield may inhale transported droplets or aerosols; in other words, the risk of infection is not low if the time when the vortex rings reach the face shield is synchronized with the inhalation period of breathing. These results also indicate that because the inflow process of the sneeze droplets into the inside of the shield has been identified, it is possible to prevent this inflow by controlling the sneeze flow. By achieving this flow control, it is possible to prevent the inflow of droplets transmitted by sneezes and coughs, even though it remains difficult to prevent the inflow of droplets floating as aerosols.

In this study, a flow simulation was performed for face shields to investigate whether the simple method of varying the edge shape of a face shield could prevent droplets from entering inside the shield.

## II. EDGE SHAPE OF A FACE SHIELD

**FIG. 1.**
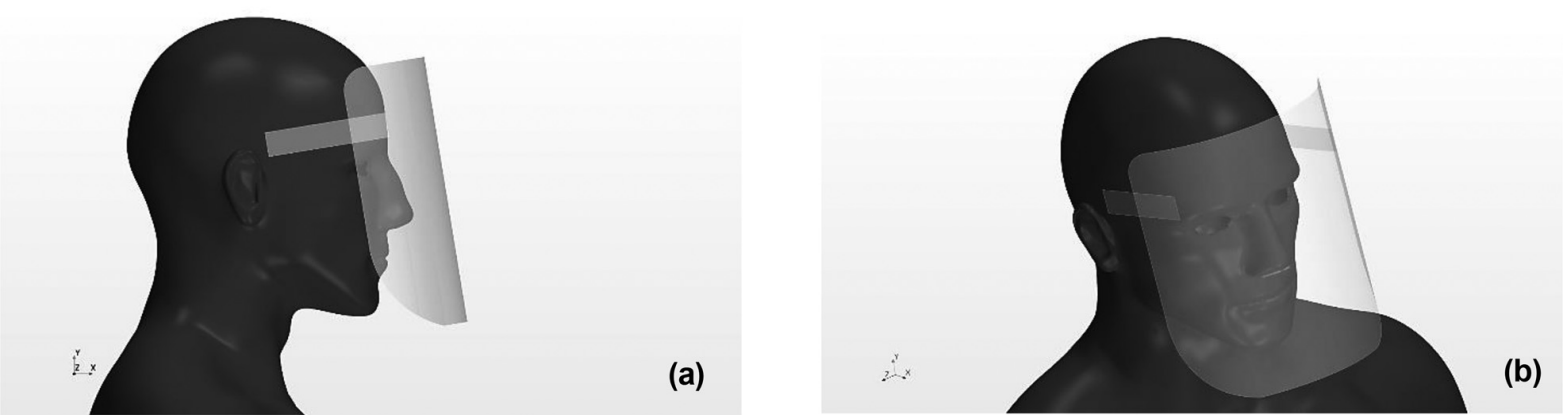
Human model wearing a regular face shield. The most popularly used shields were targeted. Figures (a) and (b) depict the side and diagonal top view, respectively.

The sneeze airflow was controlled using the edge shape of the shield, and two different designs for face shields (hereafter referred to as Type I and II) were investigated in this study. Based on our previous results obtained by simulating the airflow around a regular face shield, depicted in Fig. 1, the inflow into the inside of the shield was observed almost exclusively at the top and bottom edges of the shield, with less inflow from the sides of the shield.^29^ Therefore, the edge shape was designed only at the top and bottom edges of the shield to control the sneeze airflow.

**FIG. 2.**
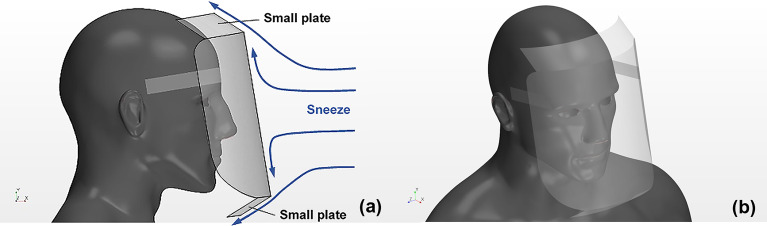
Human model wearing the Type I face shield. Small plates are attached to the upper and lower ends of the shield, inclining backward against the shield surface. Figures (a) and (b) depict the side and diagonal top view, respectively.

Figure 2 depicts the side and diagonal top view of the Type I shield. Small simple plates that had a length of 40 mm were mounted at the top and bottom edges of the shield to prevent inflow. The small plates were inclined backward at an angle of 60° to the shield surface with the aim to direct the sneeze flow toward the upper side of the wearer's head and the lower side of the chest while preventing entraining flow owing to vortex rings (refer to the light blue arrows in Fig. 2). In the case of a regular face shield, it has been confirmed that droplets transported by the sneeze flow and vortex rings remain between the neck and chest of the shield user, and these droplets move inside the shield through inhalation. Therefore, if flow under the shield can be directed to the area below the chest by the small plate mounted to the lower edge of Type I, the risk of inhalation of droplets by the shield user may be considerably reduced.

**FIG. 3.**
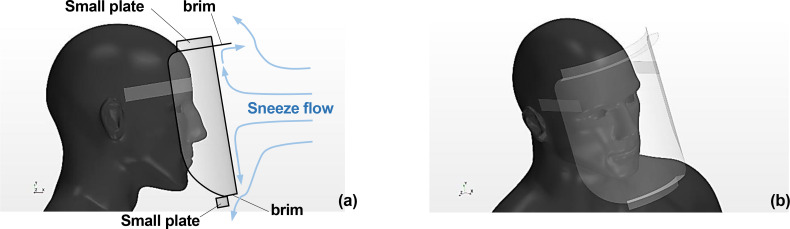
Human model wearing the Type II face shield. The upper edge of the shield has a small brim sticking forward from the shield surface and a small plate sticking upward. The lower edge of the shield has a brim sticking backward from the shield surface and a small plate sticking downward from the tip of the brim. Figures (a) and (b) depict the side and diagonal top view, respectively.

Figure 3 depicts the side and diagonal top view of the Type II shield. The upper edge of the shield had a small brim sticking forward from the shield surface and a small plate sticking upward. The length of the brim and the small plate was 20 mm. The lower edge of the shield had a brim sticking backward from the shield surface and a small plate sticking downward from the tip of the brim. The shape of the shield was aimed at controlling the propagation direction of the vortex rings by using the sneeze flow that arrives at the shield surface prior to the vortex rings (refer to the light blue arrows in Fig. 3). In other words, the sneeze flow that arrives near the top of the shield turns into a forward flow owing to the brim at the top of the shield, and this flow interferes with the vortex ring to change the propagation direction of the vortex ring upward. The brim is expected to enhance the breakdown of the vortex ring when it comes into contact with the shield surface. The small plate sticking out upward is expected to physically prevent the inflow of the vortex ring and enhance the effect of driving the vortex ring upward. The sneeze flow that arrives near the lower edge of the shield is turned into a downward flow by the small plate sticking out from the brim, and this flow changes the propagation direction of the vortex rings downward. It can also be expected to move the sneeze flow to the area below the chest. If the direction of the sneeze flow and the propagation direction of the vortex ring can be controlled as aimed, the inflow of droplets from the top of the shield and the stagnation of droplets at the bottom of the shield can be reduced, resulting in the reduced possibility of droplet inhalation.

## III. NUMERICAL METHOD

### A. Computational domain and grid

**FIG. 4.**
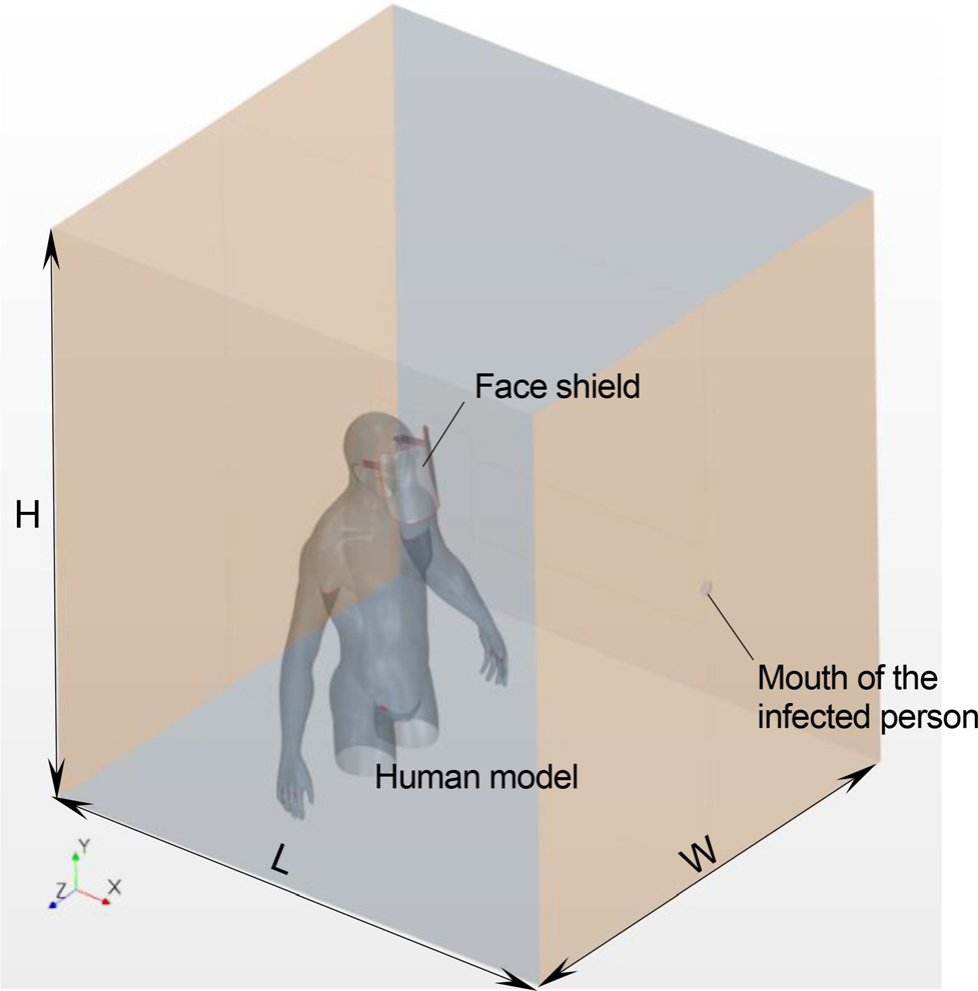
Schematic of the computational domain.

The conditions for the computational domain and grids were set according to a previous study.^29^ Figure 4 depicts a schematic of the computational domain used in the present simulation. The dimensions of this domain were 2 m × 2 m × 2.5 m in the streamwise (L), vertical (H), and width (W) directions, respectively. Life-sized human models wearing a face shield, as shown in Figs. 1–3, were placed at the center of the computational domain. We ensured a space between the shield surface and the face of the human model to imitate the most commonly used face shields. Most face shields used in the medical field are designed with no space between the upper edge of the shield surface and the user's face. This type of shield can be evaluated by referring to the flows at the sides and the lower end of the shield used in this simulation. The distance between the face shield and face was set to an average of 25 mm. This fitting condition is similar to that in reality. The average distance between medical staff and infected patients during treatment and diagnosis was estimated to be approximately 1 m.^19,26^ Therefore, the distance between the mouth of the infected person (40 mm in diameter) and the surface of the face shield facing the infected person was set to 1 m in the simulation.

**FIG. 5.**
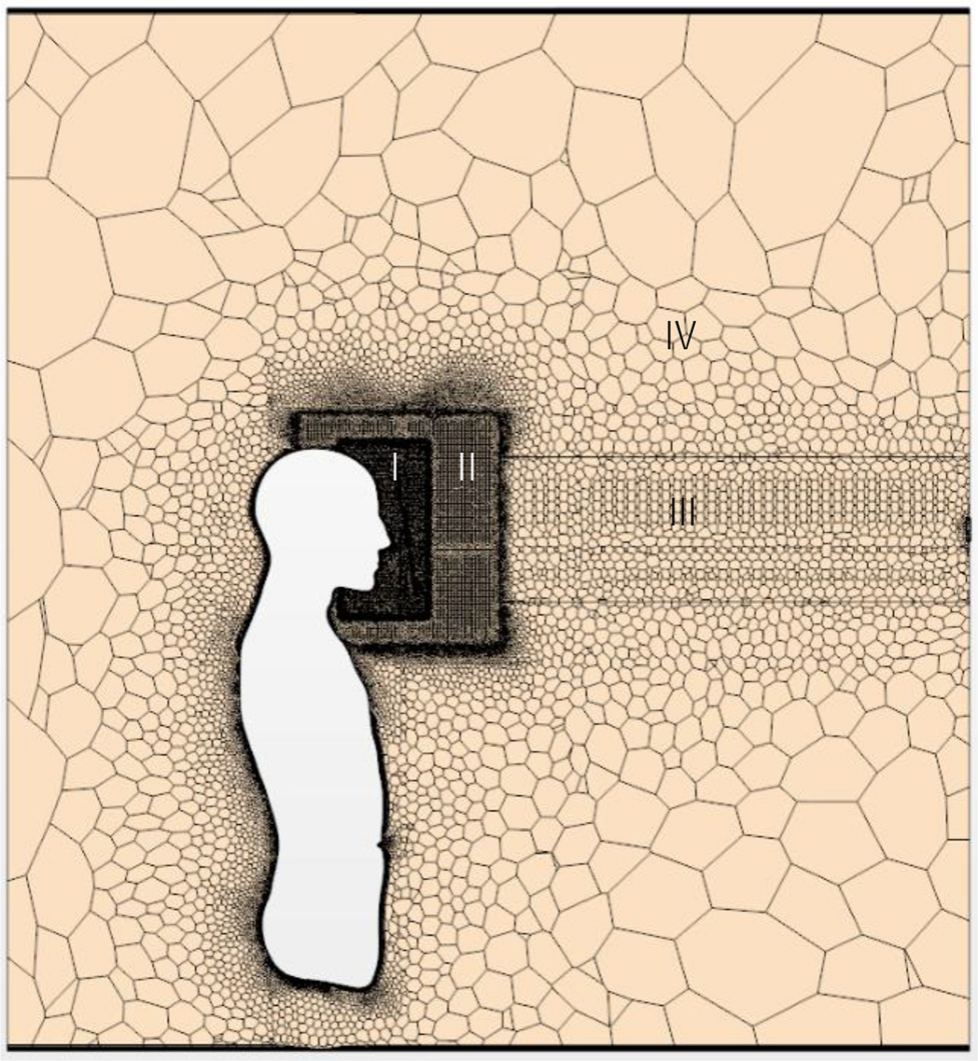
Computational grid on a vertical section in the center of the domain. The computational mesh was generated using polyhedral nonuniform structured grids. The computational domain was divided into four regions (Regions I, II, III, and IV).

The computational grids used in the simulation are presented in Fig. 5. The computational mesh was generated using polyhedral nonuniform-structured grids. As shown, the computational domain was divided into four regions (Regions I, II, III, and IV). The grid was clustered in Region I, which included the face of the human model and the face shield surface.

Moreover, the preliminary estimation of the Kolmogorov length scale of the present sneeze flow was approximately 3.5 × 10^−4^ m; therefore, this fine region consisted of grids with a size of 8.0 × 10^−4^ m (approximately double the Kolmogorov length scale). The minimum size of the grids on the surface of the shield and face was set such that it was sufficiently small to satisfy the condition y+ < 1. In the region outside Region I, the size of the grid gradually increased following a geometric progression. The estimated number of cells was 6 099 234 in Region I, 5 343 843 in Region II, 70 242 in Region III, and 1 906 316 in Region IV. Therefore, the total number of cells was approximately 13.4 × 10^6^.

### B. Boundary conditions

**FIG. 6.**
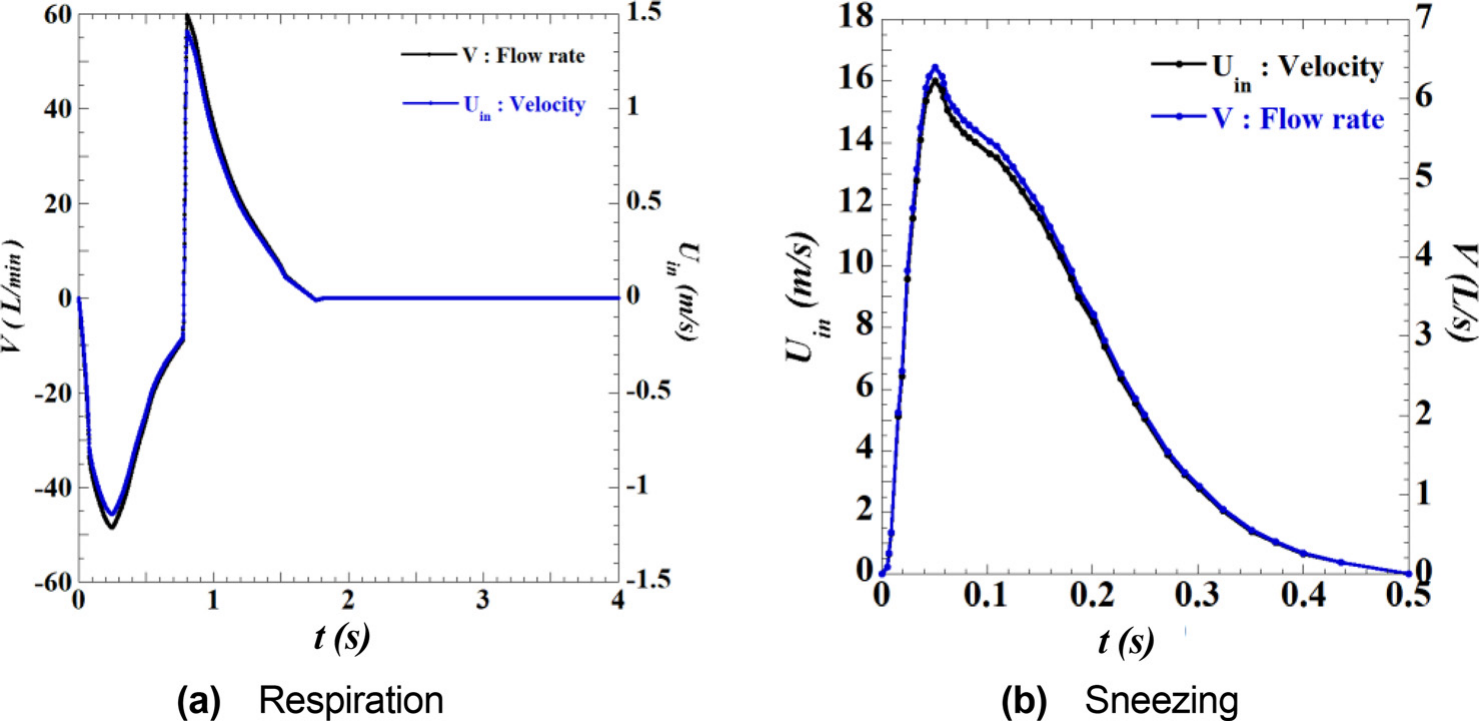
Waveform of airflow velocity and flow rate by respiration and sneezing. Figures (a) and (b) depict respiration and sneezing, respectively. Both waveforms were modeled considering a typical adult male. t indicates the time.

Figure 6 plots the velocity and flow waveforms of the airflow by respiration and sneezing.^12,27^ In Fig. 6(a), negative velocity period indicates the inhalation period, whereas a positive velocity period indicates the exhalation period. This velocity condition was applied to two elliptical inlet boundary surfaces (major diameter of 20 mm and minor diameter of 10 mm) located at the nose of the human model. This simulation assumes nasal-only respiration. The sneezing velocity condition [Fig. 6(b)] was applied to the inlet boundary surface, which simulates the mouth of an infected person. Because the temperature of the air in the mouth is approximately 32 °C (305 K),^14,24^ the temperature of the outward airflow owing to exhaling and sneezing is generally different from that of outside air. Furthermore because this temperature difference produces a buoyancy effect in the sneeze flow and vortex rings, it has been clarified that the droplets tend to be transported unevenly toward the lower end of the face shield under this condition.^29^ Therefore, it is desirable to consider the effect of the temperature difference in the simulation. Meanwhile, it is known that the transport process of droplets varies depending on the angle of the sneeze flow, which depends on the motion of the head during the sneezing, and that droplets are transported upward when the angle of the injection is higher than the horizontal direction.^12^ To evaluate the effectiveness of preventing droplets entering into the shield, which is the aim of this study, it is desirable to set the conditions such that the sneeze flow and vortex rings reach the upper and lower ends of the shield equally. For this reason, we set the air temperature at the inlet boundary to be the same as the outside temperature, which was 27 °C (300 K).

The no-slip condition was applied to the face shield surface and the human model. Specifically, in the computational domain, the aforementioned condition was applied to all boundaries except the backward boundary of the human model, for which the Neumann condition was applied such that the gradient of the physical value was zero.

### C. Flow solver

In the present simulation, a large eddy simulation was performed to simulate the vortex structure generated by the sneeze flow near the face shield. The code solved the filtered three-dimensional compressible Navier–Stokes equations using a fully implicit scheme with the finite-volume method.^28^ The convection flux was evaluated using the bounded central difference scheme. We found that this scheme turned into a first-order upwind scheme when the convection boundedness criterion was not satisfied. In other cases, it was similar to the central difference scheme, which is second-order accurate. The subgrid eddy viscosity was modeled using the wall-adapting local-eddy viscosity model.^30^

To obtain a time-accurate solution, the second-order backward difference is applied at each time step until the residual of the solution becomes less than 1 × 10^−6^. The time step size of the implicit scheme was set to 5 × 10^−4^ s, corresponding to a Courant number of 1.0. The computational fluid dynamics code StarCCM+ (version 12.06.011) was employed, and the computations were performed on two Intel Xeon (E5–2699 v4; total number of cores = 44) processors. The computational time required for our simulation experiment was approximately 1 week.

The validation of the simulation needs to be verified by comparing the results with the experimental results of the flow around the face shield, but this experiment has not yet been conducted. As a substitute, the simulation results of coaxial jets using the computational conditions and solver presented above have been compared with the experimental results.^31^ The sneeze flow and the coaxial jets are similar in that the vortex ring structures are generated by the high-velocity flow and the mixing of the flow field is enhanced by the breakdown of the vortex ring structures. Therefore, the verification results will be useful. From the validation results, it was confirmed that the vortex structures of the jets obtained by the simulations were in good agreement with the experimental results, and the length of the potential core of the inner circular jet agreed with the experiments within 8% accuracy.^31^ Experimental validation of the flow around the face shield under the same conditions as the present simulation will be conducted in the near future.

## IV. RESULTS AND DISCUSSION

### A. Effect of the edge design of the shield on the flow characteristics

We conducted a flow simulation under the following conditions. Medical staff wearing regular, Type I, and Type II face shields are exposed to a sneeze, whose source is at a distance of 1 m, from the front. To simulate the risk of infection for medical staff, the time of the staff's inhalation should be synchronized with the time when droplets emitted by sneezing reach the surroundings of the face shield. Therefore, in this simulation, the start time of inhalation was set to 0.25 s after the start of sneezing, considering the time spent by the droplets to arrive.

**FIG. 7.**
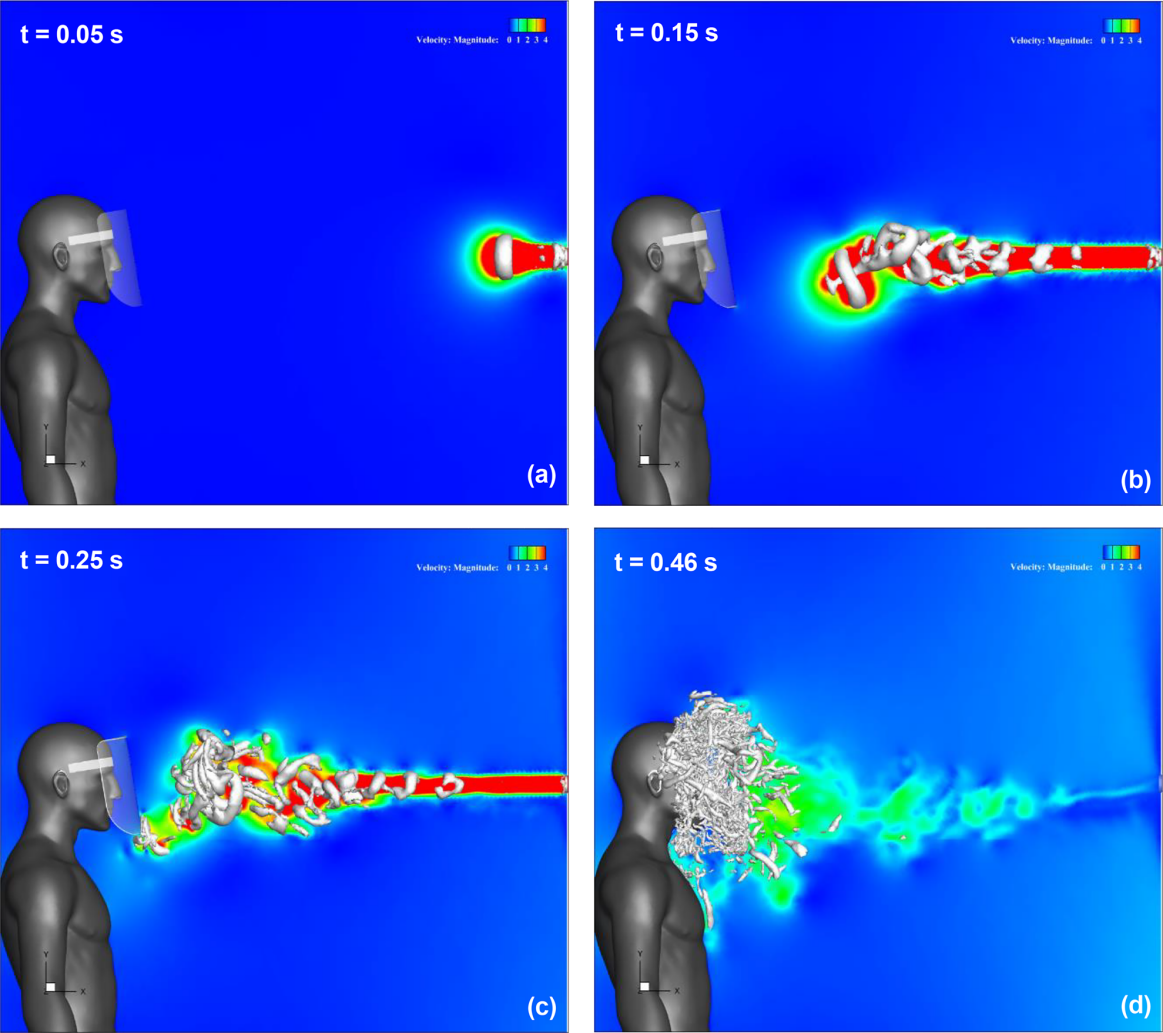
Results for a regular face shield. The streamwise velocity distribution along the vertical cross section at the center of domain and the three-dimensional vortex structure are shown. Figures (a), (b), (c), and (d) depict the results at t = 0.05 s, 0.15 s, 0.25 s, and 0.46 s, respectively. The color contours represent the magnitude of the velocity, and the isosurfaces represent the vortex structures. The vortex structure is represented by the isosurfaces of the second invariant of the velocity gradient tensor (Q-criterion). t indicates the elapsed time from the start of the sneeze. Multimedia view: https://doi.org/10.1063/5.0044367.1
10.1063/5.0044367.1

Figure 7 (Multimedia view) depicts the streamwise velocity distribution and evolution of the three-dimensional vortex structure in the regular face shield. The vortex structure is represented by the isosurfaces of the second invariant of the velocity gradient tensor (Q-criterion). Figure 7(a) (Multimedia view) represents t = 0.05 s after the start of sneezing. The sneeze generates a jet-like high-velocity flow downstream of the mouth of the infected person, and a vortex ring is generated. Furthermore, this vortex ring moves forward owing to its self-induced velocity and changes direction downward, as shown in Fig. 7(b) (Multimedia view). Subsequently, it reaches the bottom of the face shield at t = 0.25 s, as shown in Fig. 7(c) (Multimedia view). At this time, a high-velocity region can be observed at the lower end of the shield owing to the rotation of the vortex ring. Behind this vortex ring, several other vortex rings were generated [Fig. 7(b) (Multimedia view)], which moved directly forward and gradually broke down [Fig. 7(c) (Multimedia view)] and reached the front surface of the shield [Fig. 7(d) (Multimedia view)]. The flow in the vicinity of the face shield is strongly disturbed by this breakdown of the vortex rings. The flow and vortex ring behaviors presented above are different from the results obtained when the temperature difference is considered.^29^ Under the condition that the temperature difference between the air in the mouth and the outside air is 5 °C, it has been observed that the leading vortex ring tends to move upward because of the effect of buoyancy owing to the temperature difference. In the present condition without a temperature difference, the leading vortex ring moved downward owing to the effect of longitudinal vortices generated by the turbulence of the vortex rings, but the other vortex rings moved almost directly.^32,33^ Although such differences owing to the effects of buoyancy on the flow characteristics and the behavior of the vortex rings are observed, the face shield is covered with a vortex structure, and the high-velocity area caused by the sneeze flow is also observed from the neck to the chest of the shield user; therefore, stagnation of the inflow inside the shield and the droplets under the shield are considered to have occurred.

**FIG. 8.**
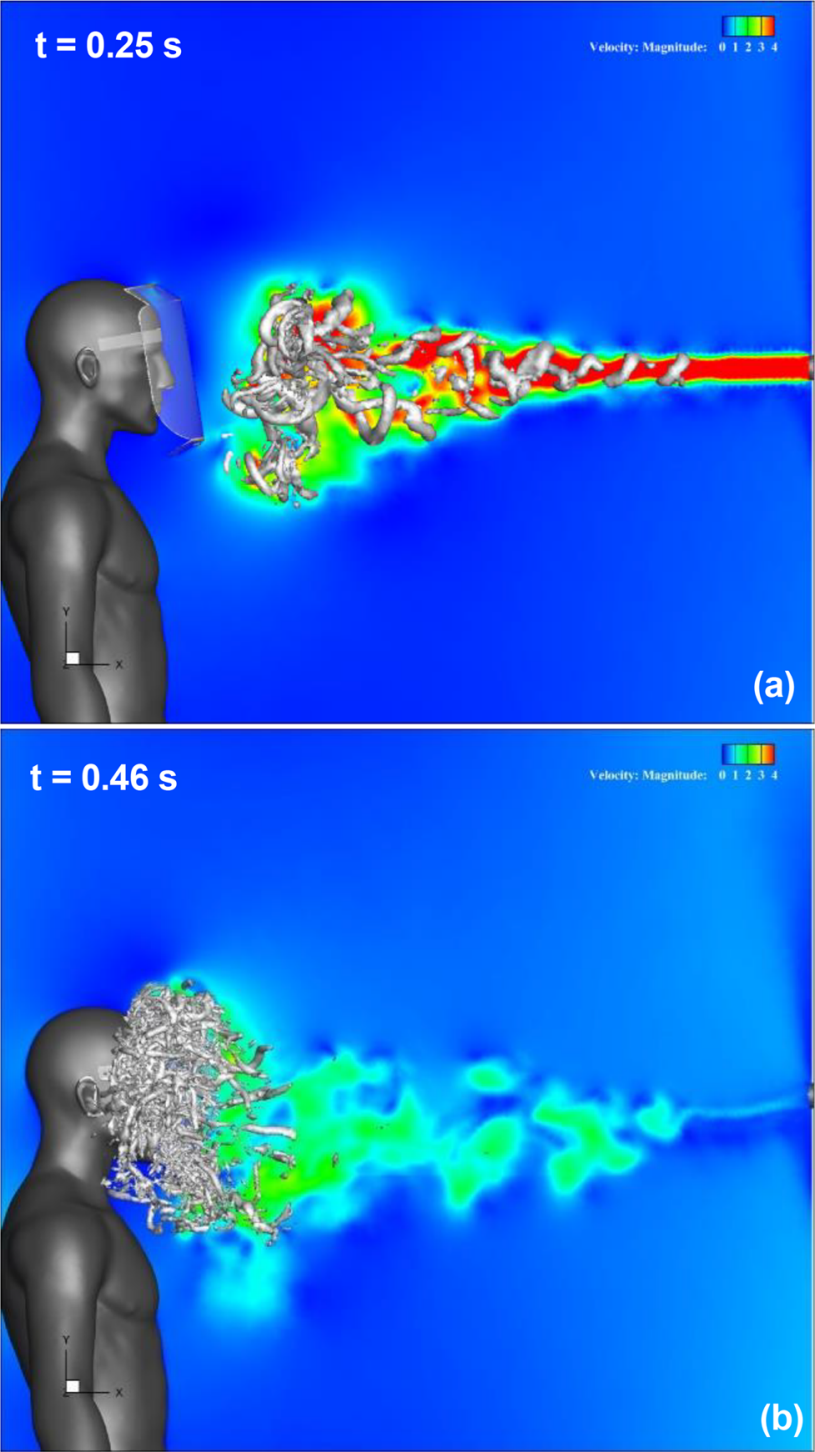
Results for Type I face shield. The streamwise velocity distribution along the vertical cross section at the center of the domain and the three-dimensional vortex structure are shown. Figures (a) and (b) depict the results at t = 0.25 s and 0.46 s, respectively. The color contours represent the magnitude of the velocity, and the isosurfaces represent the vortex structures. t indicates the elapsed time from the start of the sneeze. Multimedia view: https://doi.org/10.1063/5.0044367.2
10.1063/5.0044367.2

Figure 8 (Multimedia view) depicts the streamwise velocity distribution and evolution of the three-dimensional vortex structure in the Type I face shield. The leading vortex ring generated by the sneeze flow at t < 0.25 s moves downward and reaches the vicinity of the bottom of the face shield at t = 0.25 s, as shown in Fig. 8(a) (Multimedia view). The position of the vortex ring and the behavior of its breakdown are slightly different from those of the regular shield. This difference is observed when the vortex ring makes contact with the shield surface; therefore, we consider that it is due to the difference in the shape of the shield edge. Behind this vortex ring, several other vortex rings were generated [Fig. 8(a) (Multimedia view)]. These rings moved directly forward and gradually broke down and reached the front surface of the shield [Fig. 8(b) (Multimedia view)]. With the focus now on the area below the neck of a shield user, it can be confirmed that the high-velocity area caused by the sneeze flow, which was observed for a regular shield, is not observed, and the sneeze flow spreads slightly downward of the shield. This difference seems to be caused by the shape of the lower edge of the shield.

**FIG. 9.**
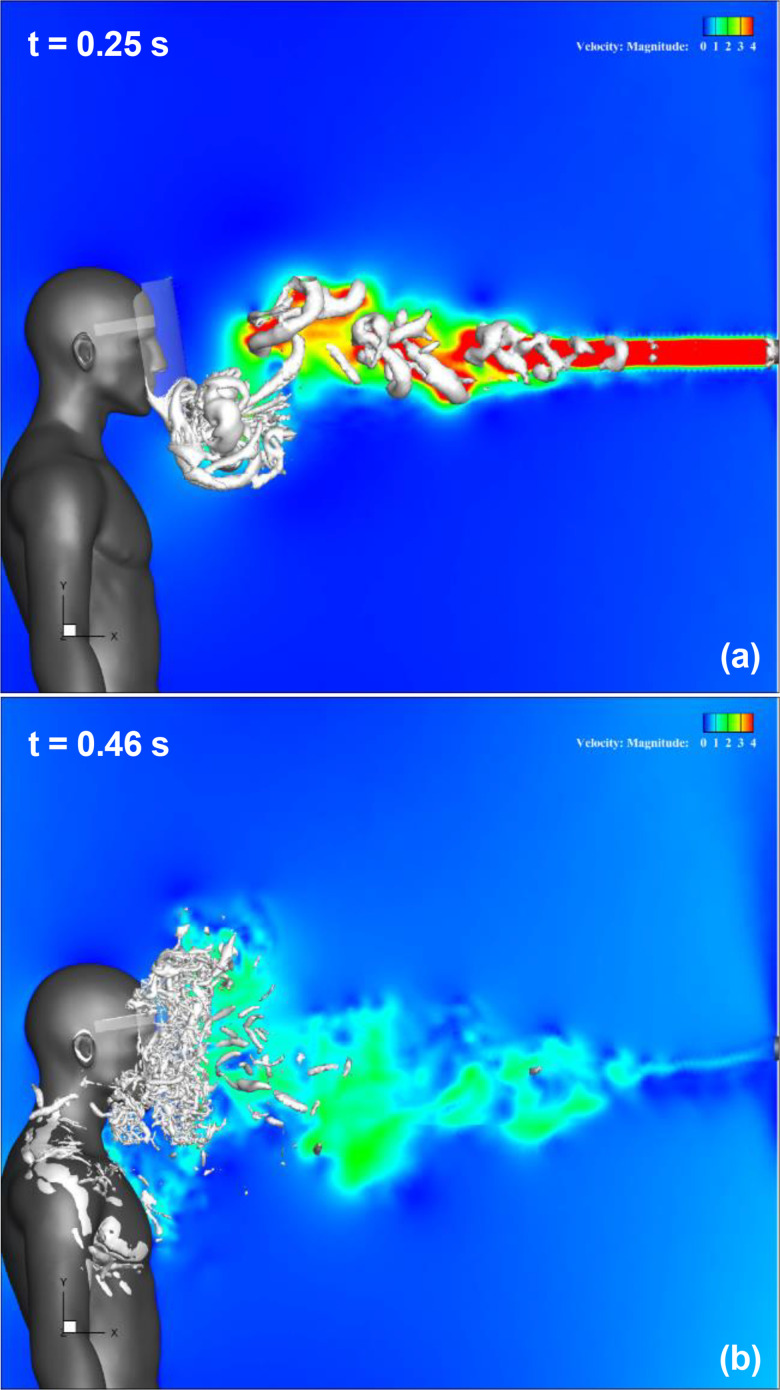
Results for Type II face shield. The streamwise velocity distribution along the vertical cross section at the center of the domain and the three-dimensional vortex structure are shown. Figures (a) and (b) depict the results at t = 0.25 s and 0.46 s, respectively. The color contours represent the magnitude of the velocity, and the isosurfaces represent the vortex structures. t indicates the elapsed time from the start of the sneeze. Multimedia view: https://doi.org/10.1063/5.0044367.3
10.1063/5.0044367.3

Figure 9 (Multimedia view) depicts the streamwise velocity distribution and evolution of the three-dimensional vortex structure in the Type II face shield. The leading vortex ring generated by the sneeze flow moves downward and reaches the vicinity of the bottom of the face shield at t = 0.25 s, as shown in Fig. 9(a) (Multimedia view). The position of the vortex rings and the behavior of their breakdown are slightly different from those of the regular and Type I shields owing to the unsteady characteristics of the vortex ring. These rings moved directly forward and gradually broke down and reached the front surface of the shield [Fig. 9(b) (Multimedia view)]. Focusing on the area below the neck of a shield user, it can be confirmed that the high-velocity area caused by the sneeze flow, which was observed for a regular shield, is not observed, and the sneeze flow spreads slightly downward of the shield. This difference seems to be caused by the shape of the lower edge of the shield. Further focus on the upper area of the shield reveals that the vortex structure extends upward in front of the shield, which is different from those of regular and Type I shields. This difference seems to be caused by the shape of the upper edge of the shield.

The comparison of the flow characteristics and the vortex structure around the face shield with two types of edge shapes confirms that the flow characteristics around the face shield can be controlled by the shape of the shield edge. Furthermore, the Type I shield is effective in controlling the flow on the lower side of the shield, and the Type II shield is effective in controlling the flow on the upper side of the shield. To confirm the effect of the shield edge shape on the flow characteristics in further detail, the time evolutions of the velocity distributions around the shields are compared.

**FIG. 10.**
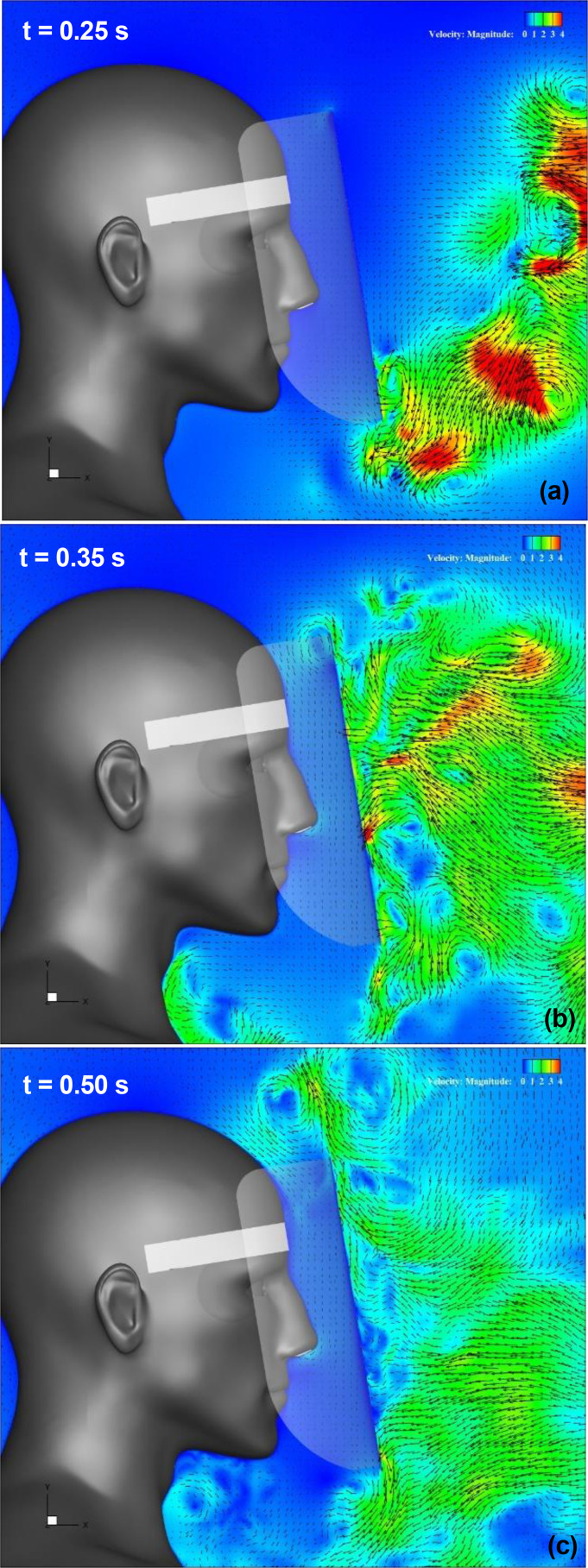
Results for a regular face shield. Vertical projections of the velocity vectors along the vertical cross section at the center of the domain. Figures (a), (b), and (c) depict the results at t = 0.25 s, 0.35 s, and 0.50 s, respectively. The arrows and colors represent the velocity vectors and the magnitudes of the velocities, respectively. t indicates the elapsed time from the start of the sneeze. Multimedia view: https://doi.org/10.1063/5.0044367.4
10.1063/5.0044367.4

Figure 10 (Multimedia view) depicts the distribution of the vertical projection velocity vectors along the vertical cross section at t = 0.25, 0.35, and 0.5 s for the regular face shield. At t = 0.25 s [Fig. 10(a) (Multimedia view)], when the leading vortex ring touches the bottom of the shield and the shield wearer's begins to inhale, the low-velocity flow from the outside of the shield to the inside begins to be generated by the swirling flow of the vortex at the bottom edge of the shield. At t = 0.35 s [Fig. 10(b) (Multimedia view)], when the high-velocity region caused by the trailing vortex rings makes contact with the shield surface, the entraining flow into the shield is generated at the upper edge of the shield. This time synchronizes with the timing when the shield wearer's inhalation flow rate is increased, and a high-velocity area near the wearer's nose can be observed, indicating that the wearer is inhaling air from the top of the shield, which may also have affected the increase in the entrainment flow velocity. At the bottom of the shield, the upward flow generated by the flow passing through the lower edge of the shield and impinging on the chest of the shield user reaches the neck. It has been observed that this upward flow tends to cause stagnation of droplets.^29^ The entraining flow at the upper edge of the shield and the upward flow at the bottom edge continue at t = 0.5 s [Fig. 10(c) (Multimedia view)].

**FIG. 11.**
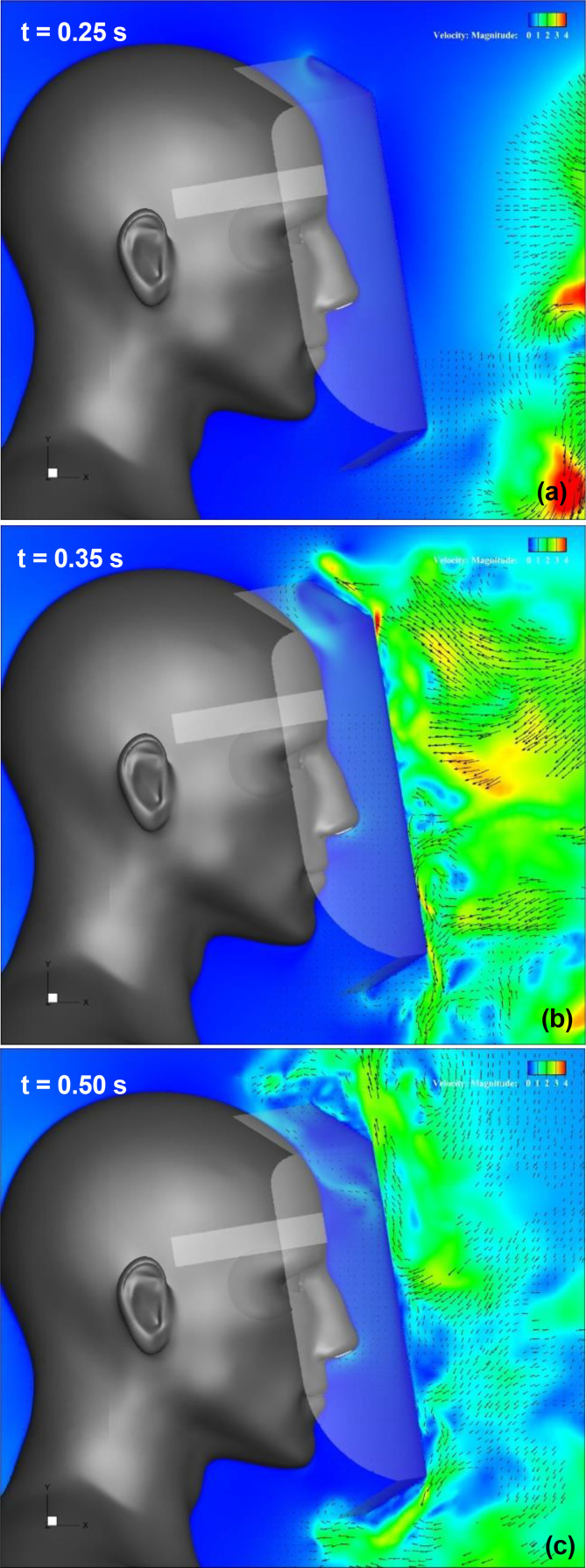
Results for a Type I face shield. Vertical projections of the velocity vectors along the vertical cross section at the center of the domain. Figures (a), (b), and (c) depict the results at t = 0.25 s, 0.35 s, and 0.50 s, respectively. The arrows and colors represent the velocity vectors and the magnitudes of the velocities, respectively. t indicates the elapsed time from the start of the sneeze. Multimedia view: https://doi.org/10.1063/5.0044367.5
10.1063/5.0044367.5

Figure 11 (Multimedia view) depicts the distribution of the vertical projection velocity vectors along the vertical cross section in the Type I face shield. At t = 0.25 s [Fig. 11(a) (Multimedia view)], the low-velocity flow begins to form along the surface of the small plate mounted on the bottom edge of the shield, induced by the rotating flow of the vortex ring moving in the vicinity of the bottom edge. Because the rotating flow of the vortex ring induces not only fluid on the surface of the small plate but also flow inside the shield, the fluid inside the shield moves slowly downward, resulting in the generation of an entraining flow from the outside to the inside at the top end of the shield. At t = 0.35 s [Fig. 11(b) (Multimedia view)], the sneeze flow impinging on the front surface of the shield is divided into upper and lower parts and flows along the shield surface; an upward and backward flow along the small plate is generated at the upper part of the shield. In addition, the velocity of the entrained flow generated at the top edge of the shield has increased. In contrast, the downward flow along the shield surface is separated at the connection between the shield body and the small plate, and flow along the small plate is not observed. The flow direction of the separated flow is almost vertically downward such that the attachment point of the flow becomes lower than that of the wearer's chest. This point is different from that of the regular face shield. At t = 0.5 s [Fig. 11(c) (Multimedia view)], the entering flow at the top edge of the shield extends downward and reaches the area near the eye. At the bottom edge of the shield, the direction of the flow changes from vertically downward to along the small plate, but no upward flow from the chest to the neck of the wearer is generated.

**FIG. 12.**
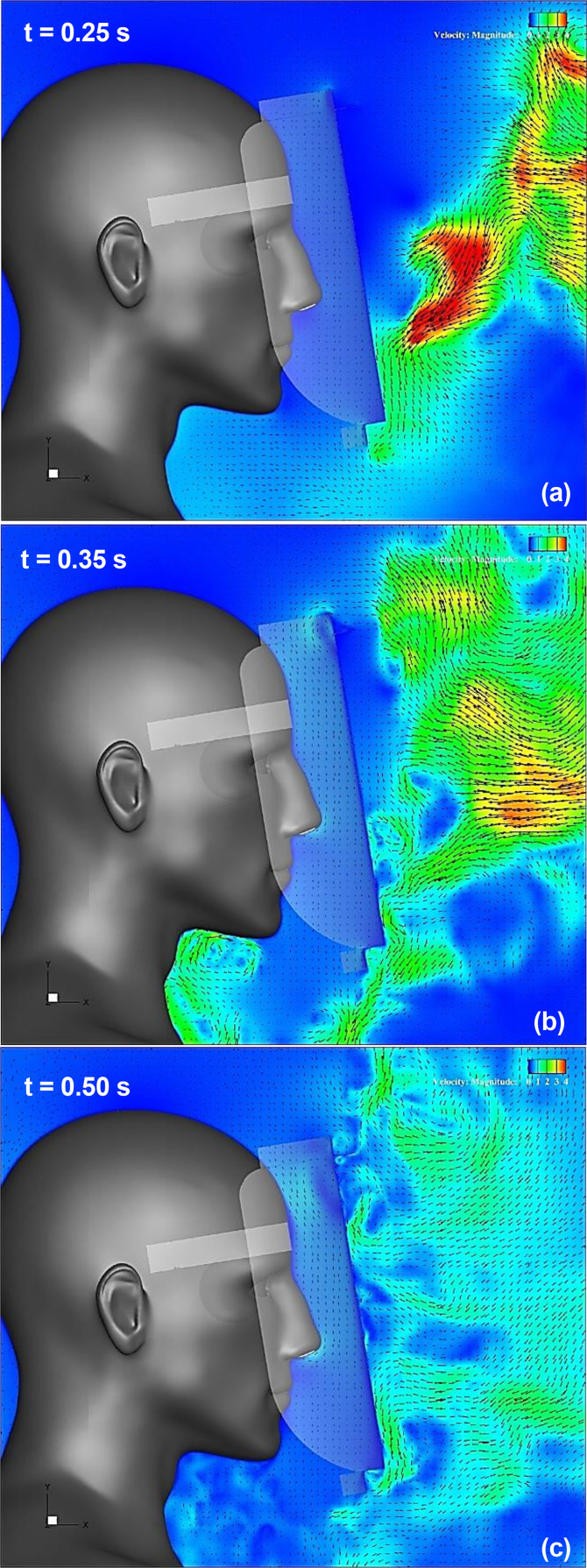
Results for a Type II face shield. Vertical projections of the velocity vectors along the vertical cross section at the center of the domain. Figures (a), (b), and (c) depict the results at t = 0.25 s, 0.35 s, and 0.50 s, respectively. The arrows and colors represent the velocity vectors and the magnitudes of the velocities, respectively. t indicates the elapsed time from the start of the sneeze. Multimedia view: https://doi.org/10.1063/5.0044367.6
10.1063/5.0044367.6

Figure 12 (Multimedia view) depicts the distribution of the vertical projection velocity vectors along the vertical cross section of the Type II face shield. At t = 0.25 s [Fig. 12(a) (Multimedia view)], the rotating flow of the vortex ring at the bottom edge of the shield generates a flow along the lower part of the shield. At this time, no flow enters the inside of the shield, and there is no flow toward the chest of the wearer. At t = 0.35 s [Fig. 12(b) (Multimedia view)], the upward flow generated by the impact on the chest of the shield user after passing through the bottom edge of the shield reaches the neck. The brim and the small plate were designed to turn the direction of the flow passing near the bottom edge to the vertical downward direction and to inhibit the upward flow generated near the chest, i.e., they were factors in the generation of stagnant droplets, but it seems that the effect was not sufficient. In contrast, at the upper end of the shield, the flow generated by the vortex rings and sneeze flow is not observed, and the only flow into the inside of the shield is the flow owing to respiration. This point is different from that of the regular face shield. These flow characteristics are maintained at t = 0.5 s [Fig. 12(c) (Multimedia view)]. Further, a weak rotating flow is generated at the upper and lower sides of the brim mounted at the top edge of the shield. On the upper side of the brim, the rotating flow indicates a clockwise rotation, whereas the lower side indicates a counterclockwise rotation. This rotating flow is considered to contribute to the inhibition of flow generation owing to vortex rings and sneeze flow.

**FIG. 13.**
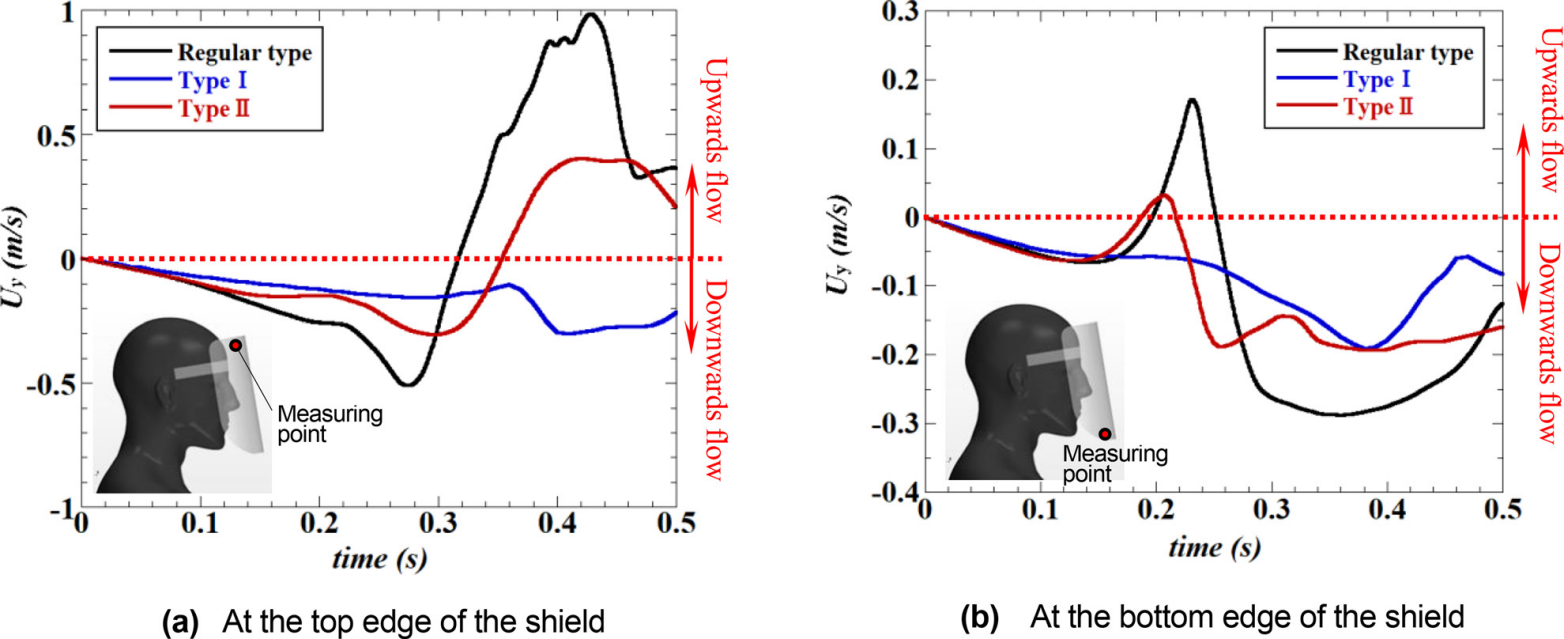
Time evolution of the vertical (Y-direction) velocity near the top [Fig. (a)] and bottom [Fig. (b)] edges of the face shield. Velocity measurements were conducted at two positions on a vertical section through the center of the shield: 5 mm inside the inner surface of the shield, and 5 mm below the top edge and 5 mm above the bottom edge of the shield (these positions are indicated by the red dots shown in each figure). (a) At the top edge of the shield. (b) At the bottom edge of the shield.

Figure 13 depicts the time evolution of the vertical (Y-direction) component of the velocity near the top and bottom edges of the face shield. Velocity measurements were conducted at two positions on a vertical section through the center of the shield: 5 mm inside the inner surface of the shield, and 5 mm below and 5 mm above the top and bottom edges of the shield. From the velocity evolution near the top edge, it can be observed that, in the regular face shield, the entering velocity from the outside to the inside of the shield (at this time, the flow from the top to the bottom is generated inside the shield) gradually increases with the approach of the sneeze flow, and the velocity reaches its maximum just after t = 0.25 s when the sneeze flow reaches the bottom edge of the shield. Afterward, when the high-velocity flow induced by the vortex rings reaches the top edge of the shield, the entering velocity decelerates rapidly and changes into the outflow (the upward flow). This variation in velocity indicates the entraining flow due to the vortical structure generated by the roll-up of the separated flow from the top of the shield [Figs. 10(b) and 10(c) (Multimedia views)]. In other words, the high velocity of this upward flow indicates that the entraining flow is strong. In the Type I shield, the low-velocity inflow will always be generated, independent of the approaching sneeze flow or vortex rings [This is also visible in Fig. 11 (Multimedia view)]. This velocity value is much lower than that of the regular shield, indicating that the small plate mounted on the top edge of the shield effectively controls the inflow. In the Type II shield, the variation in velocity is similar to that in the regular shield, but the velocity is reduced to less than half, indicating that the shape of the top edge of the regular shield effectively controls the inflow [This is also visible in Fig. 12 (Multimedia view)].

From the velocity evolution near the bottom edge of the shield, it can be observed that an inflow (upward flow) from the outside to the inside of the shield is generated around t = 0.25 s when the sneeze flow reaches the regular face shield [see also Fig. 10(a) (Multimedia view)]. Subsequently, the inflow changes into the outflow, which seems to be caused by the upward flow generated near the neck of the shield wearers [see also Figs. 10(b) and 10(c) (Multimedia views)]. In the Type I shield, the flow is always toward the bottom edge of the shield (i.e., outflow). This is caused by the constant inflow from the top edge [see Fig. 11 (Multimedia view)], and it can be observed that no entraining flow is generated at the bottom edge. In the Type II shield, as in the top edge, it can be observed that the variation in velocity is similar to that of the regular shield, but that the velocity value is reduced to less than half [see Fig. 12 (Multimedia view)].

**FIG. 14.**
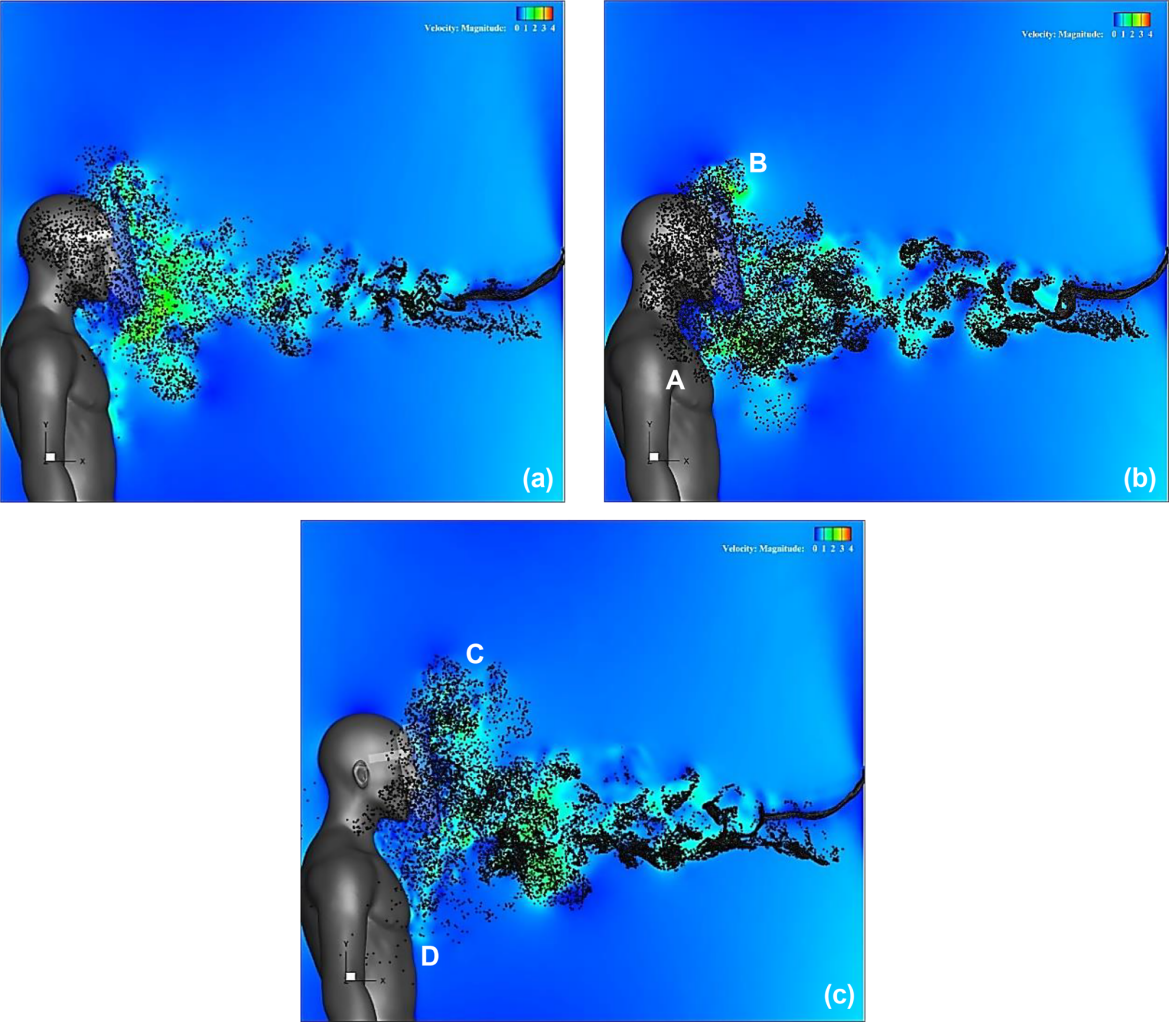
Distribution of aerosol-sized particles. Figures (a), (b), and (c) depict the results for a regular, Type I, and Type II face shield, respectively. Fifty particles were injected into the sneeze flow every 0.01 s at the inlet boundary, which simulates the mouth of an infected person. Multimedia views: https://doi.org/10.1063/5.0044367.7
10.1063/5.0044367.7; https://doi.org/10.1063/5.0044367.8
10.1063/5.0044367.8; https://doi.org/10.1063/5.0044367.9
10.1063/5.0044367.9

A comparison between the velocity distributions indicates that the flow characteristics around the face shield can be controlled by the shape of the shield edge. The effect of this flow control is expected to sufficiently prevent the entrance of droplets. Figure 14 (Multimedia view) depicts the results of particles spreading at t = 0.54 s when the inhalation flow rate is close to the maximum. Generally, the simulation of the trajectory of these particles considers the effects of the gravitational forces, drag, droplet evaporation, droplet collapse, merger, and turbulent dispersion forces on particles.^34–37^ However, it was confirmed that droplets with a diameter of more than 60 *μ*m tend to fall down by gravity rather than tracking the flow.^12,38^ In addition, droplets with a diameter of less than 20 *μ*m evaporate rapidly immediately after ejection and become aerosols that are easily airborne.^39^ Under the conditions of this simulation, in which the distance between the infected person and the face shield wearer was set at 1 m, droplets with a large diameter would either fall on the floor or attach to the surface of the shield, and thus would be difficult to enter inside the shield. The effect of particle size and mass is also considered to be very small because small droplets, which are easily transported by sneeze flows and vortex rings, can be regarded as aerosols. Because the purpose of this simulation was to confirm the trajectory of the droplets when they travel with the flow, it was assumed, as in the previous study,^29^ that the target droplets were small enough to trace the flow (i.e., the mass of the particles was equal to zero). Furthermore, the Runge–Kutta method was used to estimate the trajectory of the droplets. This method is equivalent to using the fluid particles in a sneeze as a sample of droplets, which would be an overestimation of the actual trajectory of the droplets; thus, the worst case of droplet inhalation could be evaluated. Fifty particles were injected into the sneeze flow every 0.01 s at the inlet boundary, which simulated the mouth of an infected person.

In the regular face shield [Fig. 14(a) (Multimedia view)], it can be observed that the droplet particles are dispersed almost homogeneously around the face of the shield user. In the Type I face shield, droplet particles are mainly dispersed on the underside of the shield, and a large number of droplets reach the wearer's chest and shoulders [location A in Fig. 14(b) (Multimedia view)]. At the top edge of the shield, droplet particles appear to be bounced up by the small plate [location B in Fig. 14(b) (Multimedia view)]. In the Type II face shield, a large number of droplets are dispersed in the upper front part of the shield, and it can be observed that the droplets are bounced up by the brim and small plate mounted on the top edge of the shield [location C in Fig. 14(c) (Multimedia view)]. Furthermore, in the lower area of the shield, droplets spread below the chest [location D in Fig. 14(c) (Multimedia view)], and the range of splashing is wider than that of the regular shield. Thus, it is clear that the splashing behavior of the droplets in the sneeze flow can be controlled by controlling the flow characteristics around the shield using the design of the face shield.

**FIG. 15.**
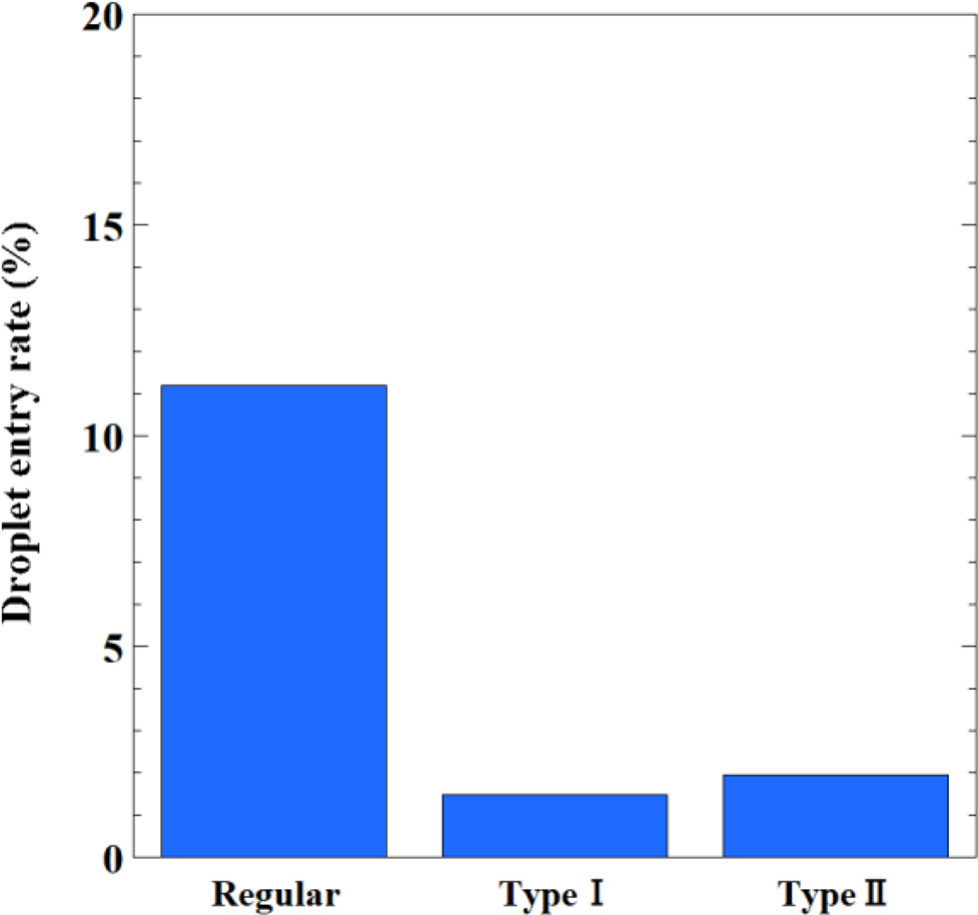
Relationship between the edge shape of the face shields and the ratio of droplets entering the inside of the shields at t = 0.54 s when the inhalation flow rate is close to the maximum. The values show the ratio of the total number of particles that were released to the number of particles that entered the inside of the shield.

Figure 15 depicts the relationship between the edge shape of the face shields and the ratio of droplets entering the inside of the shields at t = 0.54 s when the inhalation flow rate is close to the maximum. The values show the ratio of the total number of particles that were released to the number of particles that entered the inside of the shield. Regular face shields allow 11.2% of the released droplets to enter the inside of the shield, whereas Type I and Type II are able to reduce that to 1.47% and 1.94%, respectively. This reduction of entry ratio is caused by the inhibition control of entraining flow via the design of the shield edge.

From the results discussed above, it was confirmed that the flow characteristics around the face shield can be controlled by the shape of the shield edge; however, the effect of the inhibition of entraining flow varies depending on the shape. In the Type I edge shape, the entraining flow inside the shield at the bottom edge of the shield was inhibited by the mounted small plate, thereby ensuring the inhibiting effect. However, the inhibition effect of the entraining flow at the top edge was weak. To enhance this inhibition effect, it is necessary to inhibit the effect of the vortex rings and sneeze flow at the bottom edge, which induces the fluid inside the shield. In the Type II edge shape, the entrained flow inside the shield at the top edge was inhibited by the mounted brim and small plate, thereby ensuring the inhibiting effect. However, no inhibition effect of entraining flow was observed at the bottom edge. To inhibit this entraining flow, it is necessary to inhibit the effect of vortex rings and sneeze flow at the bottom edge, which induces the fluid inside the shield. To inhibit this entraining flow, the dimensions of the brim and installation angle of the small plate should to be optimized.

### B. Effect of shape sizes and mounting angles on flow characteristics

It was confirmed in Sec. IV A that the edge shape of the face shield can control the entraining flow owing to sneezing and vortex rings. This effect is expected to vary widely depending on the design parameters, such as the length and mounting angle of the edge shapes; thus, the effects of the design parameters on the flow characteristics around the face shield were examined. This evaluation was conducted for Type II shields, where the inhibition effect was expected to vary widely depending on the design parameters. The conditions examined are listed in Table I. L_1_ indicates the length of the small plate, and L_2_ indicates the length of the brim. In this study, L_1_ was fixed at 20 mm, and only L_2_ was varied (10 mm, 20 mm, and 40 mm). The angle between the small plate and the shield surface is indicated by *θ*. The angle between the brim and the shield surface was set as a perpendicular angle, as described in Sec. IV A. It should be noted that case 2 in the table was the same condition as that shown in Figs. 12 (Multimedia view), 13, 14 (Multimedia view), and 15 in Sec. IV A.

**TABLE I.** Evaluation conditions.

L_2_/L_1_	0.33	1.0	1.67
θ (deg)			
0	**Case 1**	**Case 2**	**Case 3**
35.8	**Case 4**	**Case 5**	**Case 6**

**FIG. 16.**
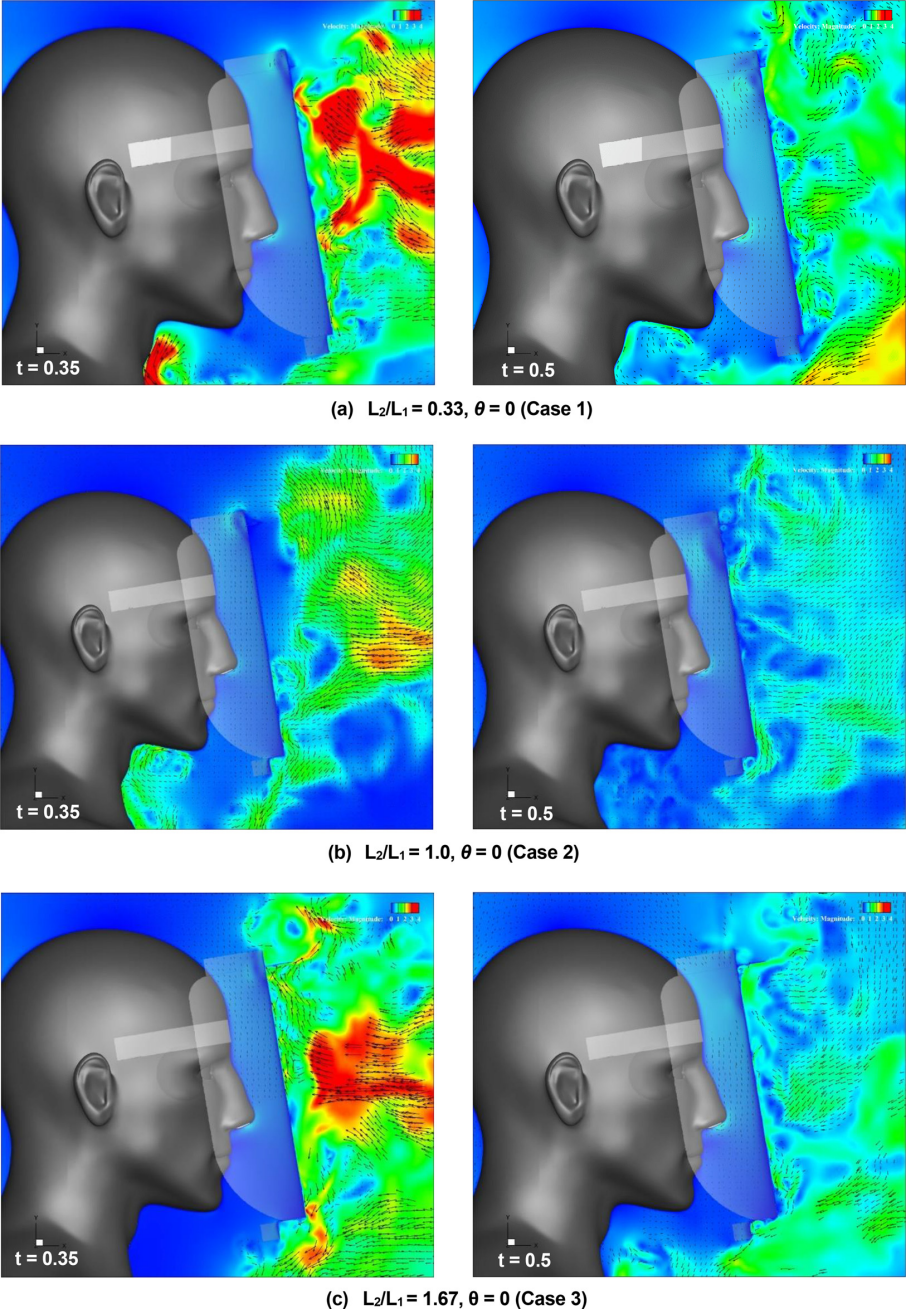
Flow characteristics around the face shield at t = 0.35 and 0.5 s for different brim lengths. Vertical projections of the velocity vectors along the vertical cross section at the center of the domain. Figures (a), (b), and (c) depict the results for the case 1, case 2, and case 3 face shield, respectively. The arrows and colors represent the velocity vectors and the magnitudes of the velocities, respectively. Multimedia views: https://doi.org/10.1063/5.0044367.10
10.1063/5.0044367.10; https://doi.org/10.1063/5.0044367.11
10.1063/5.0044367.11; https://doi.org/10.1063/5.0044367.12
10.1063/5.0044367.12

**FIG. 17.**
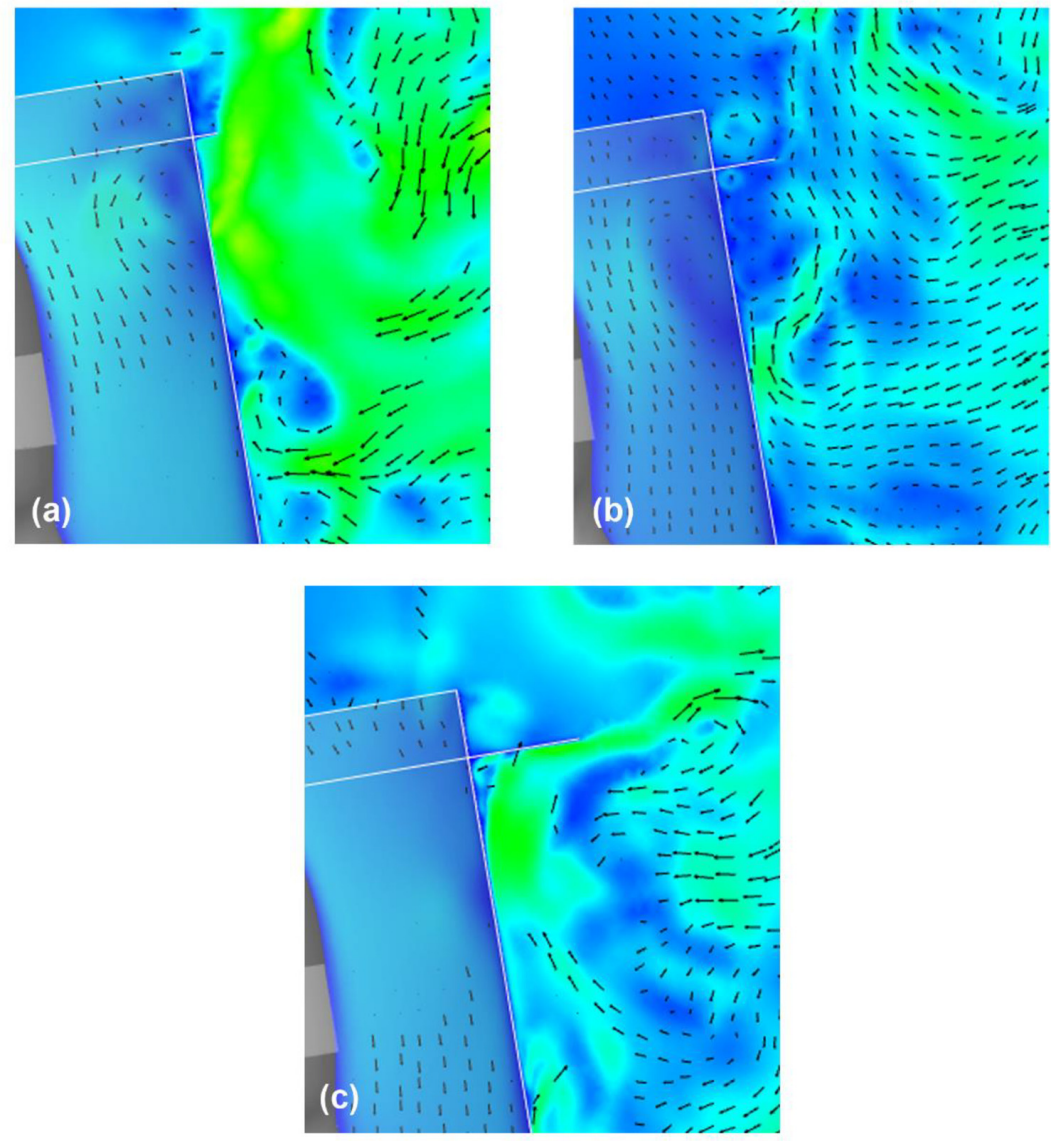
Flow characteristics around the upper end of the face shield at t = 0.5 s for different brim lengths. Figures (a), (b), and (c) depict the results for case 1, case 2, and case 3 face shield, respectively. Vertical projections of the velocity vectors along the vertical cross section at the center of the domain. The arrows and colors represent the velocity vectors and the magnitudes of the velocities, respectively.

Figure 16 (Multimedia view) depicts the distribution of the vertical projection velocity vectors along the vertical cross section at t = 0.35 and 0.5 s under different brim lengths. A comparison of the results indicates that at the top edge of the shield, the high-velocity region, which indicates the flow induced by the vortex ring, moves slightly downward in front of the shield surface as the brim length increases. Figure 17 depicts the enlarged view of the upper end of each shield. It is observed that the rotating flow generated on the upper and lower sides of the brim mounted on the top edge of the shield (the rotating flow exhibits clockwise rotation on the upper side of the brim and counterclockwise rotation on the lower side) increases as the length of the brim increases. This suggests that increasing the brim length enhances the ability to turn the sneeze flow toward the front of the shield, which contributes to the propagation of the vortex ring-induced flow toward the lower front of the shield. It is interesting to note that even in case 1, in which the brim length is the shortest (L_2_ = 10 mm), the effect of inhibiting the entrainment flow is higher than that for a regular face shield. A comparison of the flow at the bottom edge of the shield as a function of the brim length reveals that the region of high-velocity entraining flow, which represents the upward flow from the chest to the neck of the wearer, decreases as the brim length ratio (L_2_/L_1_) increases. This decrease in the high-velocity region implies that the stagnation of droplets under the neck is easily inhibited. Thus, it was confirmed that the effect of the brim length on the inhibition of the entraining flow at the top and bottom edges of the shield is significant, and to increase the inhibition effect, we should increase the length of the brim.

**FIG. 18.**
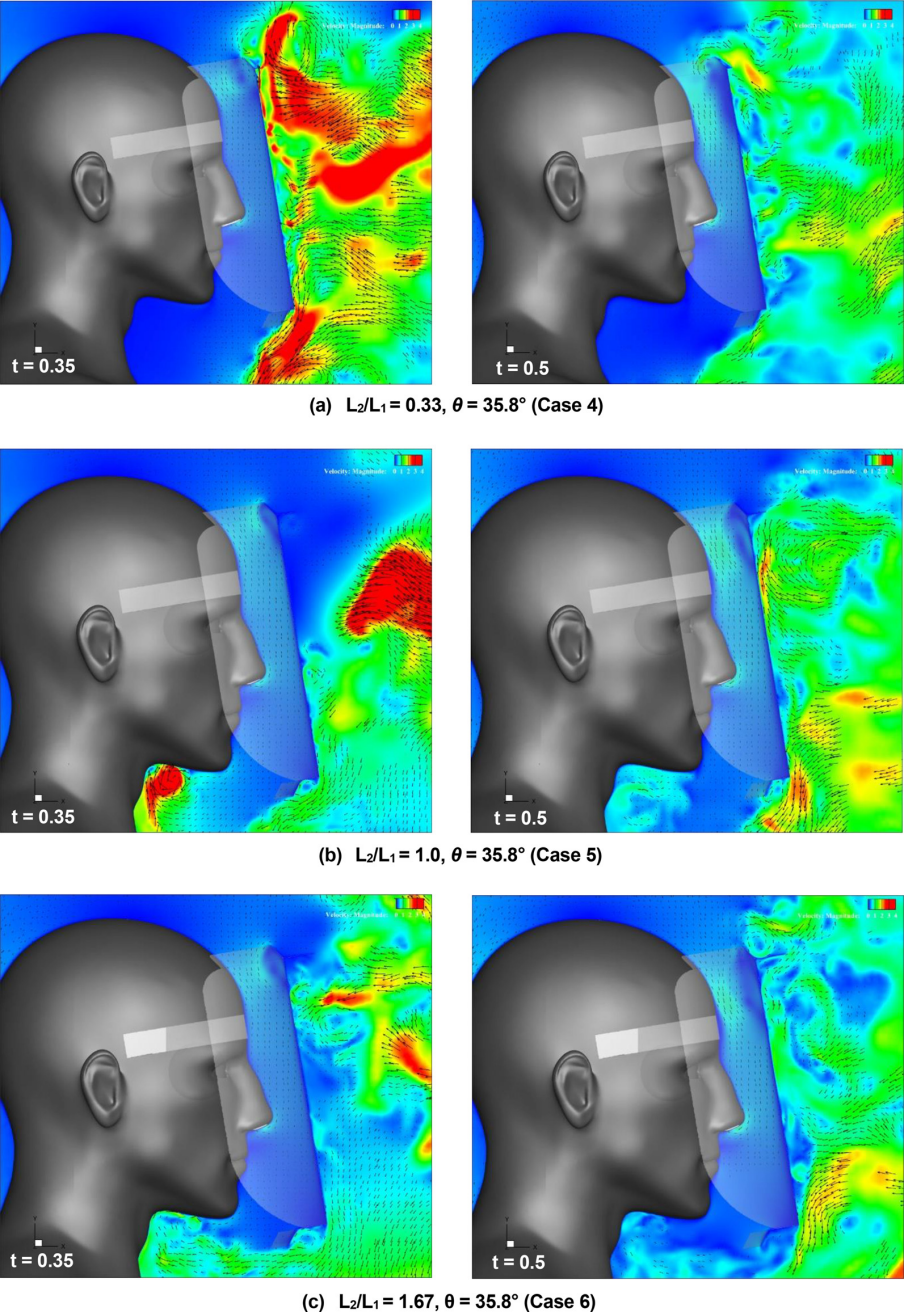
Flow characteristics around the face shield at t = 0.35 and 0.5 s for a small plate mounting angle of 35.8°. Figures (a), (b), and (c) depict the results for the case 4, case 5, and case 6 face shield, respectively. Vertical projections of the velocity vectors along the vertical cross section at the center of the domain. The arrows and colors represent the velocity vectors and the magnitudes of the velocities, respectively. Multimedia views: https://doi.org/10.1063/5.0044367.13
10.1063/5.0044367.13; https://doi.org/10.1063/5.0044367.14
10.1063/5.0044367.14; https://doi.org/10.1063/5.0044367.15
10.1063/5.0044367.15

Figure 18 (Multimedia view) depicts the distribution of the vertical projection velocity vectors along the vertical cross section at t = 0.35 and 0.5 s for a small plate mounting angle of 35.8°. Comparing the differences in the results based on the angle of inclination, at the top edge of the shield, the high-velocity region, which indicates the flow induced by the vortex ring, moves slightly downward in front of the shield surface as the brim length increases. When L_2_/L_1_ = 0.33, where the brim length is the shortest, the entraining flow owing to the vortex rings is generated at the top edge of the shield because of the inclination of the small plate. Because this edge shape is similar to that of the Type I face shield, both flow characteristics are relatively similar. However, in the other conditions for L_2_/L_1_, the entrainment flow is not generated by the inclination of the small plate, indicating that the flow characteristics are almost the same as those for θ = 0°. This indicates that the effect of the inclination angle of the small plate on the flow characteristics at the top edge of the shield decreases when the length of the brim increases. Meanwhile, comparing the differences in the results based on the angle of inclination at the bottom edge of the shield, when L_2_/L_1_ = 0.33, the region of high-velocity entraining flow is no longer observed owing to the inclination of the small plate. This result is the most effective among all the conditions examined in the present study. This flow characteristic is also similar to that of Type I, as well as the case for the top edge. However, as the value of L_2_/L_1_ increases, the region of the entraining flow is expanded by the inclination, and the inhibition effect of the entraining flow decreases. This may occur because as the brim length increases and the tip of the small plate gets close to the wearer's chest, the contact position between the sneeze flow and the body is near the chest even if the sneeze flow is along the inclination of the small plate. Thus, it was confirmed that the effect of the inclination angle of the small plate on the entraining flow characteristics around the shield was significantly enhanced at the bottom edge of the shield.

**FIG. 19.**
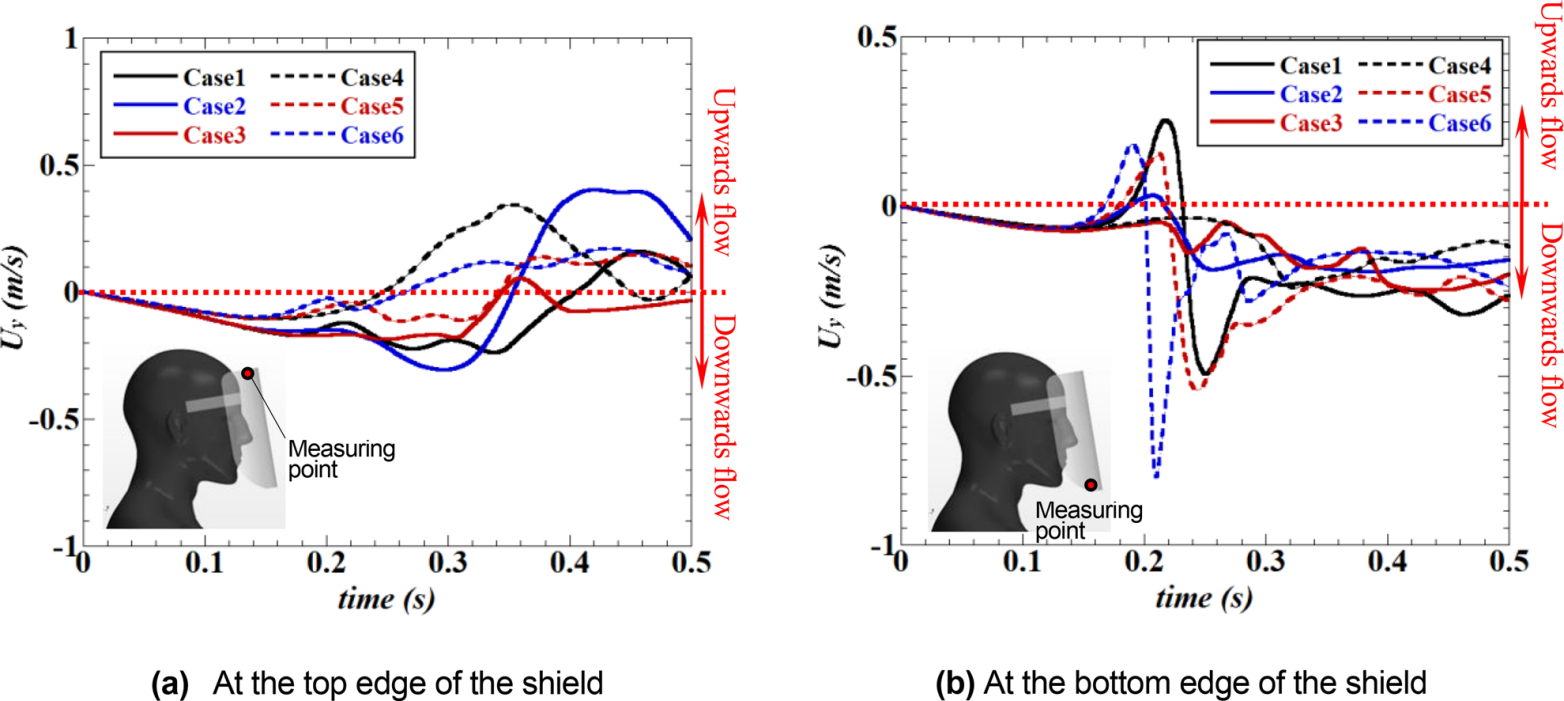
Time evolution of the vertical (Y-direction) velocity near the top [Fig. (a)] and the bottom [Fig. (b)] edges of the face shield under the conditions given in Table I. The velocity measurements were conducted at two positions on a vertical section through the center of the shield: 5 mm inside the inner surface of the shield, and 5 mm below the top edge and 5 mm above the bottom edge of the shield (these positions are indicated by red dots in each figure). (a) At the top edge of the shield. (b) At the bottom edge of the shield.

Figure 19 depicts the time evolution of the vertical (Y-direction) component of the velocity near the top and bottom edges of the face shield under all the conditions listed in Table I. At the top edge of the shield [Fig. 19(a)], as the length of the brim (L_2_) increases (solid line in the figure), the variation in the velocity caused by the entraining flow owing to sneezing and vortex rings decreases, and as the mounting angle of *θ* increases (dashed line in the figure), the variation in the velocity also decreases. Under the present conditions, the most effective inhibition of the entraining flow at the top edge of the shield was observed in Case 6, wherein the brim was the longest and the small plate was inclined. The relationship between the length of the brim and the inhibition of the entraining velocity observed at the top edge of the shield is similarly confirmed at the bottom edge of the shield [Fig. 19(b)]. As the brim length (L_2_) increases (solid line in the figure), the velocity variation decreases, and the inhibition effect of the entraining flow increases. However, this relationship changes when the small plate is inclined. When *θ* is large (dashed line in the figure), the velocity variation owing to entraining flow decreases as the length of the brim decreases, and the inhibition effect increases. Under the present conditions, the most effective inhibition of entraining flow at the bottom edge of the shield was observed in case 4, wherein the brim length was the shortest and the small plate was inclined.

**FIG. 20.**
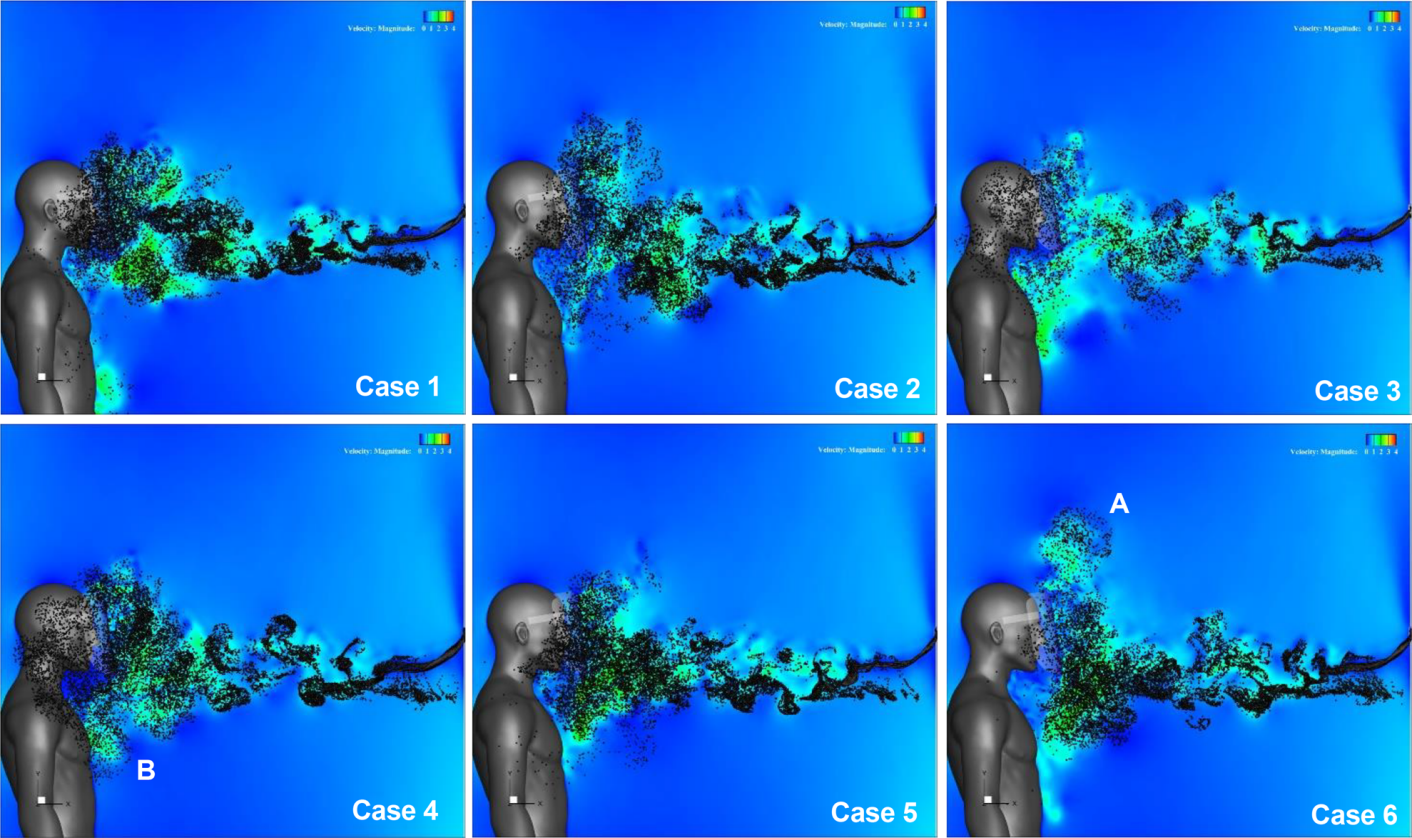
Distribution of aerosol-sized particles under the conditions given in Table I. Fifty particles were injected into the sneeze flow every 0.01 s at the inlet boundary, which simulates the mouth of an infected person. Multimedia views: https://doi.org/10.1063/5.0044367.16
10.1063/5.0044367.16; https://doi.org/10.1063/5.0044367.17
10.1063/5.0044367.17; https://doi.org/10.1063/5.0044367.18
10.1063/5.0044367.18; https://doi.org/10.1063/5.0044367.19
10.1063/5.0044367.19; https://doi.org/10.1063/5.0044367.20
10.1063/5.0044367.20; https://doi.org/10.1063/5.0044367.21
10.1063/5.0044367.21

Figure 20 (Multimedia view) presents the results of particle spreading under all the conditions shown in Table I at t = 0.54 s when the inhalation flow rate is close to the maximum. From the figure, it can be observed that the splashing distributions of the droplets vary according to the variation in the velocity distribution caused by the design conditions of the shield edge. In case 6, wherein the inhibition of entraining flow was the most effective at the top edge of the shield, the droplets spread upward in front of the shield (the area indicated by A in the figure), and the height of the spread was the highest among all the conditions. It can be confirmed that the inhibiting effect of the inflow of droplets is the highest. In case 4, wherein the inhibition effect of the entraining flow was the highest at the bottom edge of the shield, droplets spread from the lower part of the shield to the lower part of the wearer's chest (the area indicated by B in the figure), and it can be confirmed that the inhibiting effect of the inflow of droplets at the bottom edge is the most effective.

**FIG. 21.**
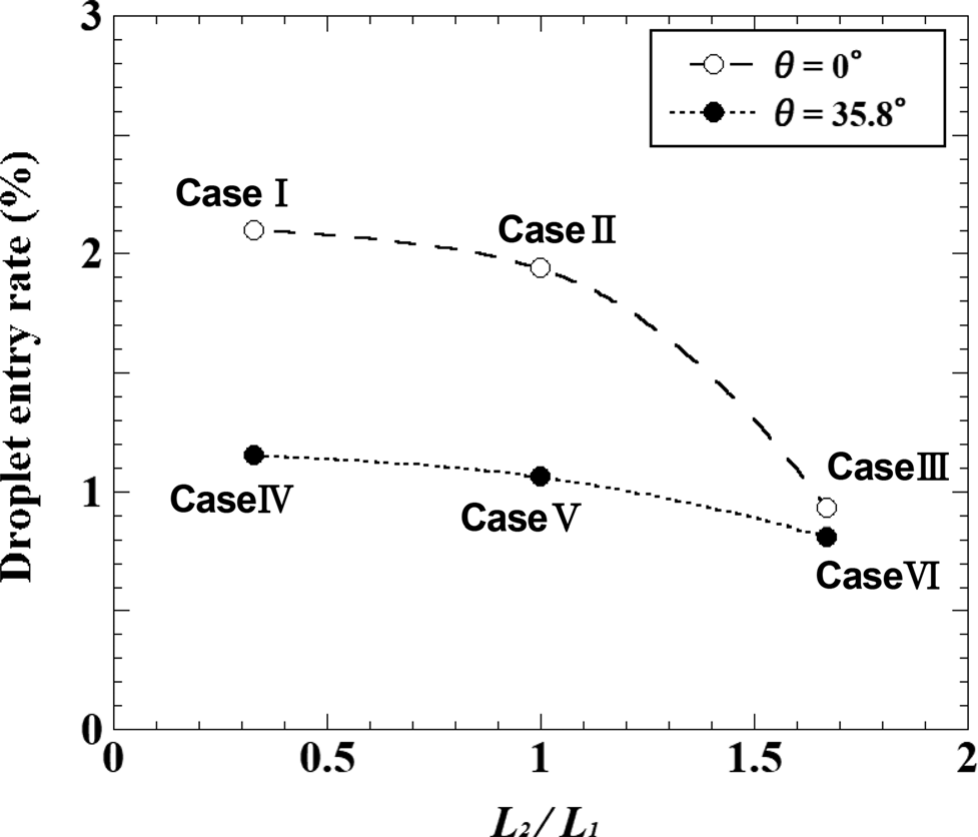
Relationship between the design parameters of the Type II face shield and the ratio of droplets entering the inside of the shields at t = 0.54 s when the inhalation flow rate is close to the maximum. The values show the ratio of the total number of particles that were released to the number of particles that entered the inside of the shield.

Figure 21 depicts the ratio of droplets entering inside the shield at t = 0.54 s, when the inlet flow rate is close to the maximum, for all the evaluation conditions shown in Table I. The number of droplets entering the inside of the shield decreased as the brim length was increased, reducing the entry ratio in half only by increasing the brim length by 20 mm. Furthermore, the rate of droplet entry can be confirmed to decrease as the mounting angle of the small plate is increased for all brim length conditions. In the above conditions, the brim length and mounting angle of the plate are set to be the same at the top and bottom edges of the shield. If the design conditions for the top edge are set under the conditions of case 6, where the effect of inflow inhibition is high at the top edge, and the design conditions for the bottom edge are set under the conditions of case 4, where the effect of inflow inhibition is high at the bottom edge, the entry rate of droplets can be the minimum.

The results of the effects of the design parameters (L_2_/L_1_ and *θ*) of the Type II face shield on the inhibition of the entraining flow around the shield indicate that the conditions for a high inhibition effect were different between the top edge and the bottom edge of the shield. At the top edge of the face shield, to improve the inhibition effect, the length of the brim should be increased, and the small plate should not be mounted at an inclined angle from the shield surface. In contrast, at the bottom edge of the face shield, to improve the inhibition effect, the length of the brim should be decreased, and the small plate should be mounted at an inclined angle from the shield surface. It should be noted that the results of the present study were obtained by examining several conditions for the design parameters for the face shield, and the possibility of inhibiting the entraining flow of the sneeze flow was only indicated for the edge shape of the shield. To determine the optimal conditions for the inhibition of entrainment, further design parameters should be studied under a wide range of conditions. We also consider that the inhibition effect of the entraining flow around the face shield varies widely depending on the angle and distance between the shield wearer and the patient. This clarification will be the subject of future work.

## V. CONCLUSIONS

A flow simulation was performed for face shields to investigate whether varying the edge shape of a face shield could prevent droplets from entering the inside of the shield. The simulation was conducted using face shields with two different types of edge shapes, which were designed based on our previous results that were obtained by simulating the flow around a regular face shield. Type I had small simple plates mounted on the top and bottom edges of the shield to physically inhibit the inflow of the sneeze. Type II had small brims sticking forward from the shield surface and small plates sticking upward and downward at the top and bottom edges of the shield. These brims and small plates were mounted for the purpose of inhibiting the entrainment flow produced by the vortex ring evolved in the sneeze flow.

It was confirmed that the flow characteristics around the face shield can be controlled by the shape of the shield edge. In the Type I shape, the entraining flow to the inside the shield at the bottom edge of the shield was inhibited by the mounted small plate, thereby ensuring the inhibiting effect. However, no inhibition effect of entraining flow was observed at the top edge. In the Type II shape, the entrained flow inside the shield at the top edge was inhibited by the mounted brim and small plate; however, no inhibition effect of the entraining flow was observed at the bottom edge.

Furthermore, the effects of the design parameters, L_2_/L_1_ and *θ*, of the Type II shield on the flow characteristics around the face shield were examined. The results indicate that at the top edge of the face shield, to enhance the inhibition effect, the length of the brim should be increased, and the small plate should not be mounted at an inclined angle from the shield surface. In contrast, at the bottom edge of the face shield, to enhance the inhibition effect, the length of the brim should be decreased, and the small plate should be mounted at an inclined angle from the shield surface.

This study focuses on revealing the possibility that the edge shape of the face shield can prevent sneeze droplets from entering the shield. For this purpose, assumptions such as neglecting the temperature difference between the sneeze and the ambient air, and neglecting the particle size distribution and mass of the droplets are made in this simulation. Ideally, these conditions should be taken into account in the simulation, and further simulations, as well as experiments, considering these conditions will be conducted as future work. Although there are some issues presented above, the results of the present study clearly indicate the possibility of inhibiting the entraining flow of the sneeze flow manipulating the edge shape of the shield. We believe that the present results can help to enhance the effectiveness in preventing infection by face shields.

## Data Availability

The data supporting the findings of this study are available from the corresponding author upon reasonable request.
